# Effect of storage conditions on salivary polyamines quantified via liquid chromatography-mass spectrometry

**DOI:** 10.1038/s41598-018-30482-x

**Published:** 2018-08-13

**Authors:** Atsumi Tomita, Masayo Mori, Kana Hiwatari, Eri Yamaguchi, Takao Itoi, Makoto Sunamura, Tomoyoshi Soga, Masaru Tomita, Masahiro Sugimoto

**Affiliations:** 1 Health Promotion and Preemptive Medicine, Research and Development Center for Minimally Invasive Therapies, Tokyo Medical University, Shinjuku, Tokyo, 160-8402 Japan; 2 Institute for Advanced Biosciences, Keio University, Tsuruoka, Yamagata 997-0052 Japan; 3 Division of Gastroenterology and Hepatology, Tokyo Medical University, Shinjuku, Tokyo 160-0023 Japan; 4 Fourth Department of Surgery, Tokyo Medical University Hachioji Medical Center, Hachioji, Tokyo 190-0998 Japan; 5 Department of Pathology Kanagawa Dental College, Post Graduate School, Yokosuka, Kanagawa 238-85850 Japan

## Abstract

Salivary polyamines are potential non-invasive tools for screening various types of cancers. For clinical use, the reproducibility of these metabolites should be evaluated under various storage conditions, including duration and temperature, to establish standard operating protocols. Polyamines and amino acids in unstimulated whole saliva were quantified via liquid chromatography-mass spectrometry. Concentrations of time course samples were analysed after short-term storage for up to 240 min and long-term storage for up to 8 days under various storage conditions. As expected, storage at the lowest temperature (−18 °C) exerted the least pronounced effects on the quantified values in both tests. At a higher temperature, polyamines were more stable than amino acids, as evident from polyamine profiling. Addition of ethanol significantly stabilized polyamine profiles even at a higher temperature. Comparative processing of saliva revealed a minor effect of the solvent, whereas drying had a more prominent effect on polyamine profiles. Computational analyses evaluated the ability of polyamines to discriminate pancreatic cancer from controls. Repeated noise added tests were designed on the basis of the results of the storage tests; these analyses confirmed that the discriminative abilities were robust. These data contribute to the standardization of salivary storage conditions, thereby highlighting the clinical utility of saliva.

## Introduction

Molecular biomarkers available in minimally invasive biofluids enable the detection of various diseases, which could lead to early cancer detection. Certain tumour markers have been well established to detect cancers, e.g. CA19-9 and CEA are common markers indicating pancreatic cancer^1,2^. Although biomarker levels increase in patients with pancreatic cancer at advanced stages, false-positive results are frequent^3^. Therefore, new biomarkers are needed to complement these tumour markers. Recently, the advent of *omics* technologies, which allow for high-throughput detection of several novel markers simultaneously, has particularly facilitated cancer diagnosis^4^. Among various techniques, many metabolomics studies have reported the use of mass spectrometry with high sensitivity and broad range profiling^5^.

Polyamines, a class of metabolites, are elevated in several cancers, such as colorectal and pancreatic cancer^6^. In the urea cycle, arginine is converted to ornithine, and ornithine is converted to putrescine, a polyamine precursor, by ornithine decarboxylase (ODC). Putrescine is converted to spermine and spermidine, polyamines that are especially hyper-acetylated in cancer cells. The activity of ODC is negatively regulated in normal cells, while the loss of function of cancer-related genes, such as Adenomatous polyposis coli protein (*APC*), enhances polyamine synthesis and activates their acetylation^7^. The elevated polyamines in tumour tissue spread into the surrounding tissues and blood vessels^8,9^.

Elevated polyamines have been reported biological fluids of in cancer patients. A comprehensive comparison of hundreds of metabolites in blood samples of lung cancer patients and healthy subjects revealed that *N*_1_,*N*_12_-acetylspermidine provided the best contrast between specimens^10^. Polyamines reported as biomarkers for various cancers, such as colorectal^11,12^, breast^13^, pancreatic, lung^14,15^, and prostate cancer^16^ have been detected in urine, an even less invasive biofluid than blood. The least invasive biofluid, saliva, was previously assessed for biomarkers; these were present at high levels in oral, breast, and pancreatic cancers^17–19^. The concentration stability of four metabolites, including choline as an oral cancer biomarker, were analysed under various storage conditions after saliva collection^20^.

Elevated salivary polyamines have been reported in breast cancer patients^21,22^. Recently, we reported salivary polyamines in pancreatic cancer patients^23^. Although the evidence implies that these are potential detectable biomarkers, the stability of these metabolites should be evaluated such that an operational standard can be established to obtain reproducible quantified values for clinical screening of these cancers.

In this study, we evaluated the effects of storage conditions of polyamines and amino acids in salivary samples, focusing on handling protocols between saliva collection and measurement. Our comparisons included the effects of ethanol addition as a preservative, temperature, and storage duration. Time-courses of the metabolites were quantified for short (up to 240 min.) and long (up to 8 d) durations. The effect of deproteinization and drying processing protocols on salivary profiles were also evaluated. Based on these data, the expected noise caused by the storage was estimated after saliva collection. This noise was computationally added to the salivary polyamines, which were already reported as screening biomarkers for pancreatic cancer^23^. Changes in their discriminative abilities were evaluated.

## Material and Methods

### Chemicals

Liquid chromatography-mass spectrometry (LC-MS)-grade methanol and formic acid (FA) were obtained from Wako (Osaka, Japan). Heptafluorobutyric acid (HFBA) (ca. 0.5 mol/L in water) was purchased from TCI (Tokyo, Japan). A 28% ammonia solution was purchased from Kanto-Kagaku, Wako (Tokyo, Japan). Fifteen reagents were used as internal standards (IS): spermidine-d_8_ and spermine-d_8_ from Sigma Aldrich (St. Louis, MO, USA), *N*_1_-acetylspermidine-d_6_, *N*_1_,*N*_12_-diacetylspermine-d_6_, *N*_1_,*N*_8_-diacetylspermidine-d_6_*, N*_1_-acetylspermine-d_3_, and hypoxanthine-^13^C_2_,^15^N from Santa Cruz (Dallas, TX, USA), Pro-^13^C_5_,^15^N, Arg-^13^C_6_,^15^N_4_, and Lys-^13^C_6_,^15^N_2_ from ISOTEC, Sigma Aldrich, Trp-^13^C_11_,^15^N_2_, Leu-d_3_, and Phe-d_5_ from Taiyo Nippon Sanso (Tokyo, Japan), Met-^13^C_5_,^15^N from Cambridge Isotope Laboratories (Andover, MA, USA), 1,6-diaminohexane from TCI (Tokyo, Japan), and water purified with a Milli-Q system (Merck Millipore, Bedford, MA, USA).

### Experimental design

**Figure 1:**
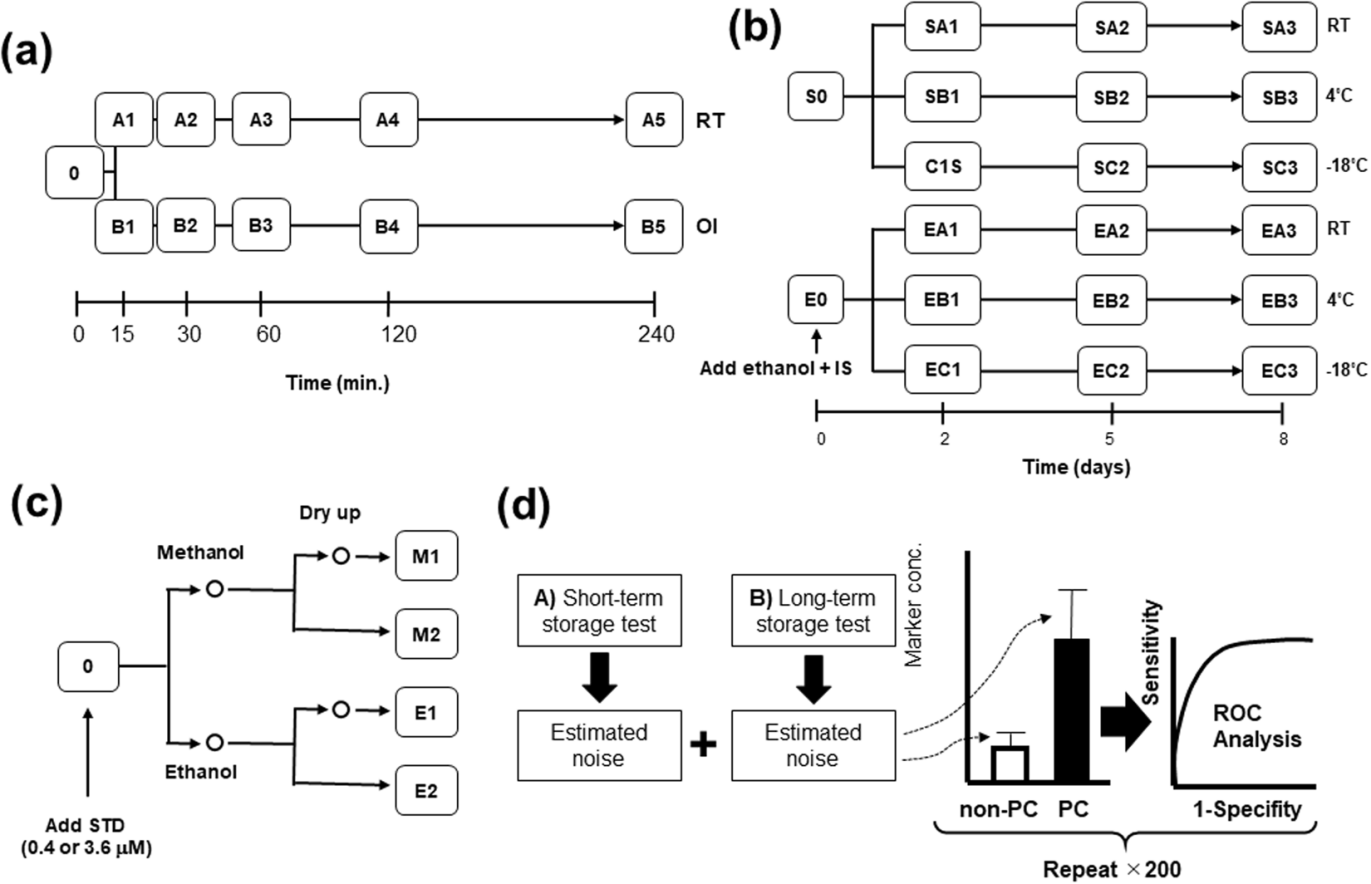
Experimental design. (**a**) Short-term storage. (**b**) Long-term storage. (**c**) Saliva sample processing. (**d**) *In silico* experiments. Sample 0 indicates the sample at time of collection. (**b**) Two types of processing were performed upon adding ethanol and internal standards (IS) (bottom) or no addition (top). Each processing was evaluated at all sampling times. (**c**) A standard mixture of 0.4 μM (sample A) and 3.6 μM (sample B) was added to the saliva samples at 0. (**c**) Saliva samples were treated with methanol or ethanol and processed with or without drying. RT: room temperature; OI: on ice. (**d**) Quantitative experiments to evaluate the effect of noise on the discrimination ability of salivary pancreatic cancer biomarkers. Based on the results of (**a**,**b**), artificial noises are added to the concentration of markers and receiver operating characteristic analyses were conducted 200 times using various random values.

Three measurement experiments were performed (Fig. 1), followed by an *in silico* experiment.

#### Short-term storage test

Saliva samples were stored at 22 °C (room temperature; RT) and on ice, and analysed at 0, 15, 30, 60, 120, and 240 min. (Fig. 1a).

#### Long-term storage test

Saliva samples were stored at −18 °C, 4 °C, and RT, and analysed at 0, 2, 5, and 8 days. Saliva samples were prepared both native and with addition of ethanol and internal standards (IS) on day 0. (Fig. 1b).

#### Solvent and dying tests

To explore potential saliva processing procedures, the effects of deproteinizing solvent and concentration by drying were evaluated (Fig. 1c). The standard mixture containing polyamines and amino acids at two different concentrations (0.4 μM and 3.6 μM) was added to the saliva samples collected from healthy subjects. These two difference concentrations were designed to include endogenous concentrations of most of the metabolites in human saliva samples listed in human metabolome database (http://www.hmdb.ca/) (Table S1) and also within the range of upper and lower limits of linearity. For solvents, methanol and ethanol were compared. The concentration process with and without drying were also compared. For both short-term and long-term storage tests, methanol addition and drying step were used. For all comparisons, four sample replicates were measured.

#### Effect of storage on discrimination capability of salivary polyamines

Data for these experiments were retrieved from a previous study^23^. Briefly, salivary samples were collected from pancreatic cancer patients (n = 39). Control samples were collected from healthy patients and those with chronic pancreatitis (n = 40). Unstimulated whole saliva samples were collected under fasting condition from 21:00 on the day prior to treatment, and any action that may affect the oral cavity, such as teeth brushing, oral care, and smoking, were abstained for at least 1 hour prior to sample collection, following a previously reported protocol^19^. Saliva samples were collected on ice and storage at −80 °C until the quantification of metabolites^23^. Polyamines that can discriminate pancreatic cancer from controls were evaluated. Randomly generated white noise whose standard deviations were determined by the results of experiments (1) and (2) were added to the original data 200 times (Fig. 1d). The areas under receiver operating characteristic (ROC) curves (AUC) were calculated. AUC values ranked at 2.5% and 97.5% are depicted.

Experiments (1) and (2) used methanol deproteinization and concentration via drying. After storage, samples were transferred to the deep freezer (−80 °C) until measurement. All samples were measured sequentially to eliminate bias resulting from MS.

To calculate the calibration curve and recover the data, all standard compounds except for *N*_1_,*N*_12_-diacetylspermine, *N*_1_,*N*_8_-diacetylspermidine, *N*_1_-acetylspermine, and hypoxanthine, were included. For the other experiments, isotope-labelled compounds of only polyamines were used to calculate the salivary polyamine concentrations. For the other metabolites, 1,6-diaminohexane was used.

This study was approved by the ethics committee of Tokyo Medical University (approval no. 1560, 30 September 2010). Written informed consent was obtained from all patients and from volunteers who agreed to donate saliva, in accordance with the tenets of the Declaration of Helsinki.

### Processing of human saliva with drying step

Human saliva (10 μL) was mixed with methanol (90 μL) containing 149.6 mM ammonium hydroxide (1% (v/v) ammonia solution) and 0.9 μM internal standards (d_8_-spermine, d_8_-spermidine, d_6_-*N*_1_-acetylspermidine, d_3_-*N*_1_-acetylspermine, d_6_-*N*_1_,*N*_8_-diacetylspermidine, d_6_-*N*_1_,*N*_12_-diacetylspermine, hypoxanthine-^13^C,^15^N, 1,6-diaminohexane, ^13^C,^15^N-Arg, ^13^C,^15^N-Lys, ^13^C,^15^N-Met, ^13^C,^15^N-Pro, ^13^C,^15^N-Trp, d_3_-Leu, and d_5_-Phe). After centrifugation at 15,780 × *g* for 10 min at 4 °C, the supernatant was transferred to a fresh tube and vacuum-dried. The sample was reconstituted with 90% methanol (10 μL) and water (30 μL), then vortexed and centrifuged at 15,780 × *g* for 10 min at 4 °C. The 1 μL of supernatants were injected into the LC-MS system.

### Processing of human saliva without drying step

Human saliva (10 µL) was mixed with methanol (30 μL) containing 149.6 mM ammonium hydroxide (1% (v/v) ammonia solution) and 2.5 μM internal standards (d_8_-spermine, d_8_-spermidine, d_6_-*N*_1_-acetylspermidine, d_3_-*N*_1_-acetylspermine, d_6_-*N*_1_,*N*_8_-diacetylspermidine, d_6_-*N*_1_,*N*_12_-diacetylspermine, hypoxanthine-^13^C,^15^N, and 1,6-diaminohexane). After centrifugation at 15,780 × *g* for 10 min at 4 °C, the 30 μL supernatant was transferred to a fresh tube. The sample was mixed with 50 μL water and centrifuged at 15,780 × *g* for 10 min at 4 °C. The 1 μL of supernatants were injected into the LC-MS system.

### LC-MS condition

The LC system 1290 Infinity (Agilent Technologies, Santa Clara, CA, USA) comprised a HiP sampler, quaternary pump, and column compartment. The setup was slightly different for triple quadrupole MS (QQQ-MS) and time-of-flight MS (TOF-MS). Chromatographic separation was performed using an ACQUITY BEH C18 column (2.1 mm i.d. × 50 mm, 1.7 μm; Waters, Milford, MA, USA) at 40 and 48 °C for QQQ-MS and TOF-MS, respectively. The mobile phase comprised solvent A (0.1% formic acid and 1.5 mM HFBA in water) and solvent B (1.5 mM HFBA in methanol), delivered at a flow rate of 0.4 mL/min and 0.3 mL/min for QQQ-MS and TOF-MS, respectively. The gradient elution conditions are listed in Table S2. The total run times for LC-MS analysis were 10 and 11 min per sample for QQQ-MS and TOF-MS, respectively. An ACQUITY UPLC BEH C18 VanGuard Pre-Column (2.1 mm i.d. × 5 mm, 1.7 μm; Waters, Milford, MA, USA) was solely used for TOF-MS.

MS detection was conducted on Agilent Technologies 6460 triple quadrupole and Agilent technologies G6230B time-of-flight for QQQ-MS and TOF-MS systems, respectively. QQQ-MS system was used for the reproducibility and linearity experiments and TOF-MS system was used for the other experiments. The samples were analysed in positive ion mode. Instrument parameters were set as follows: drying gas temperature, 275 °C and 350 °C for QQQ and TOF, respectively; drying gas flow, 13 L/min; nebulizer, 55 psig; Vcap, 3500 V. The specific multiple reaction monitoring (MRM) transitions, fragmentor voltage, and collusion energy (CE) were optimized for each compound analysed (Table S3). TOF-MS values were set as follows: Fragmentor, 125 V; Skimmer1, 90 V; OctopoleRFPeak, 200 V; mass range, 50–1000 *m/z*; scan rate, 1.00 spectra/s. Agilent MassHunter Qualitative Analysis and Quantitative Analysis software were used for data processing, including the MassHunter Optimizer and the Dynamic Multiple Reaction Monitoring Mode (DMRM) software (version B.08.00, Agilent Technologies).

### Software

Analyses were conducted using R (ver. 3.4.3, R Foundation for Statistical Computing, Vienna, Austria)^24^, GraphPad Prism (ver 7.03, GraphPad Software Inc., San Diego, CA, USA), and JMP Pro (ver. 13.2.0, SAS Institute Inc., Cary, NC, USA).

## Results

**Figure 2:**
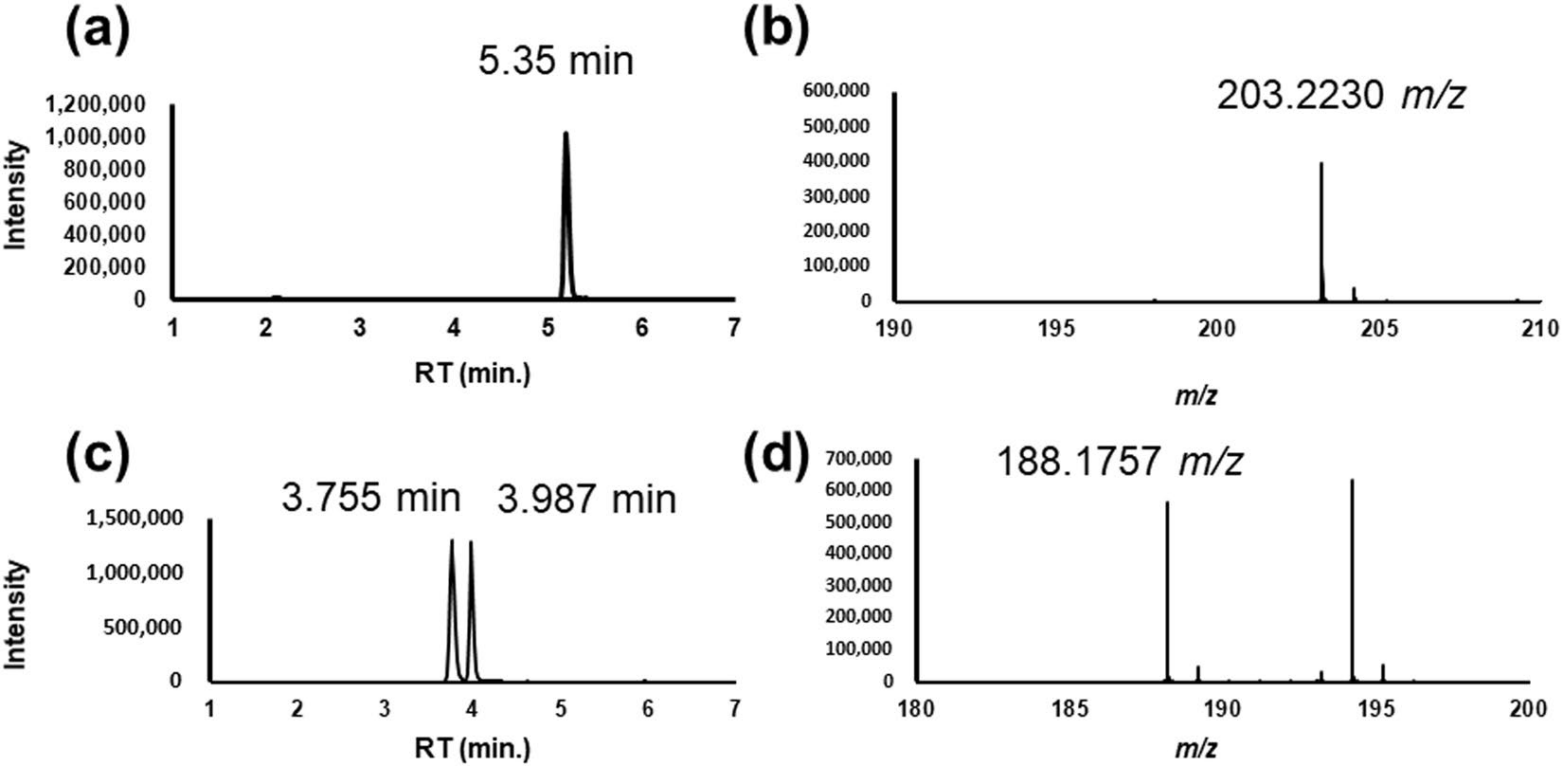
Examples of extracted ion chromatograms (EIC) and mass spectra. (**a**) EIC (203.2230 *m/z*) and (**b**) mass spectrum (5.35 min) of spermine. (**c**) EIC (188.1757 *m/z*) of *N*_1_-acetylspermidine (3.755 min) and *N*_8_-acetylsperimidine (3.987 min). (**d**) Mass spectrum (188.1757 *m/z*) of *N*_1_-acetylspermidine (3.755 min).

Polyamines, amino acids, and several other metabolites were quantified in this study. Extracted ion chromatograms (EIC) and mass spectra of spermine, *N*_1_-acetylspermidine, and *N*_8_-acetylsperimidine are depicted in Fig. 2. The recovery and variations (relative standard deviation, RSD %) of the concentrations in the standard compound mixture are listed in Table S4. The calibration curves and the range of concentrations are described in Table S5. Except for hypoxanthine (*R*^2^ = 0.9991) and histidine (*R*^2^ = 0.9993), all metabolites showed good linearity (*R*^2^ > 0.9997) and a 1.1% coefficient of variations.

### Short-term storage

**Figure 3:**
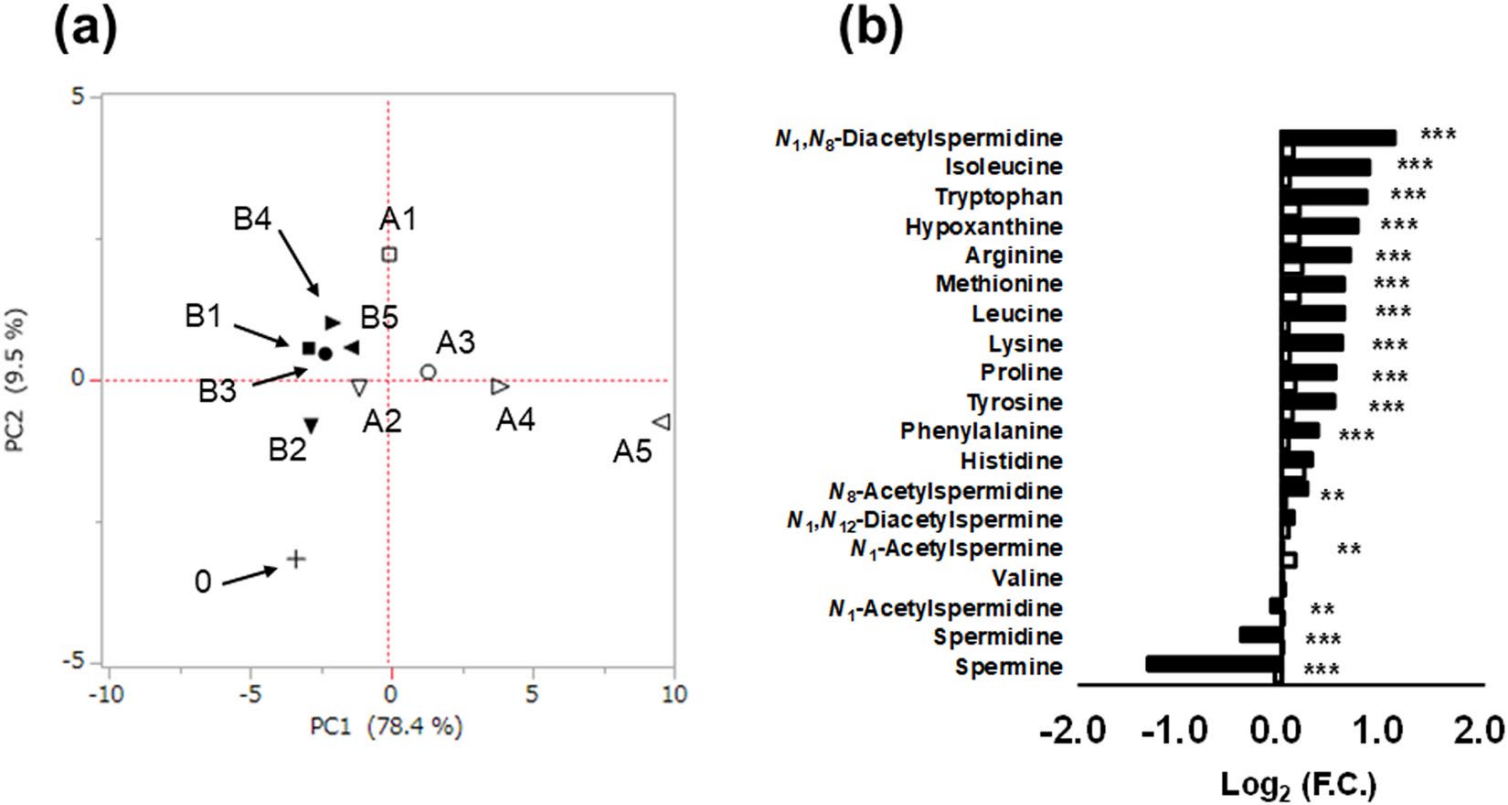
Metabolite concentration change in the short-term storage test. (**a**) Score plots of principal component (PC) analysis. The PC1 and PC2 indicated the first and the second PC. The contributions for PC1 and PC2 were 78.4 and 9.5%, respectively. Labels corresponded to Fig. 1a. (**b**) Log_2_ values of fold change of metabolite concentrations after 240 min. Black and white indicate storage at room temperature (RT) and on ice (OI), respectively, corresponding to A5/0 and B5/0 in Fig. 1a. **P < 0.01 and *P* < 0.001 (Student’s t-test, both tail).

Figure 3a and Fig S1a showed score and loading plots of principal component (PC) analysis (PCA), respectively. The contribution ratio of PC1 (78.4%) was larger than that of PC2 (9.5%), and therefore the difference in PC1 was large in metabolic profiles, i.e. metabolite concentration pattern. In respect to PC1 values, compared to the collected samples (plots labelled as 0), the samples on ice (OI) (plots labelled with B) showed a smaller difference than the samples at RT (plots labelled with A). Figure 3b depicts the comparison of salivary metabolite concentration between 0 and 240 min. The averaged fold change (F.C.) of all metabolites was 1.07 and 1.26 on ice (OI) and at RT, respectively. Increases and reductions were consistent between temperatures for 18 metabolites. Spermine, spermidine, and *N*_1_-acetylspermidine decreased while *N*_1_,*N*_8_-diacetylspermidine increased at RT. Except for valine, all amino acids showed a smaller change than *N*_8_-acetylspermidine, *N*_1_,*N*_12_-diacetylspermine, and *N*_1_-acetylspermine. Time-courses of metabolite showing larger F.C. at RT are depicted in Fig. S2.

### Long-term storage

**Figure 4:**
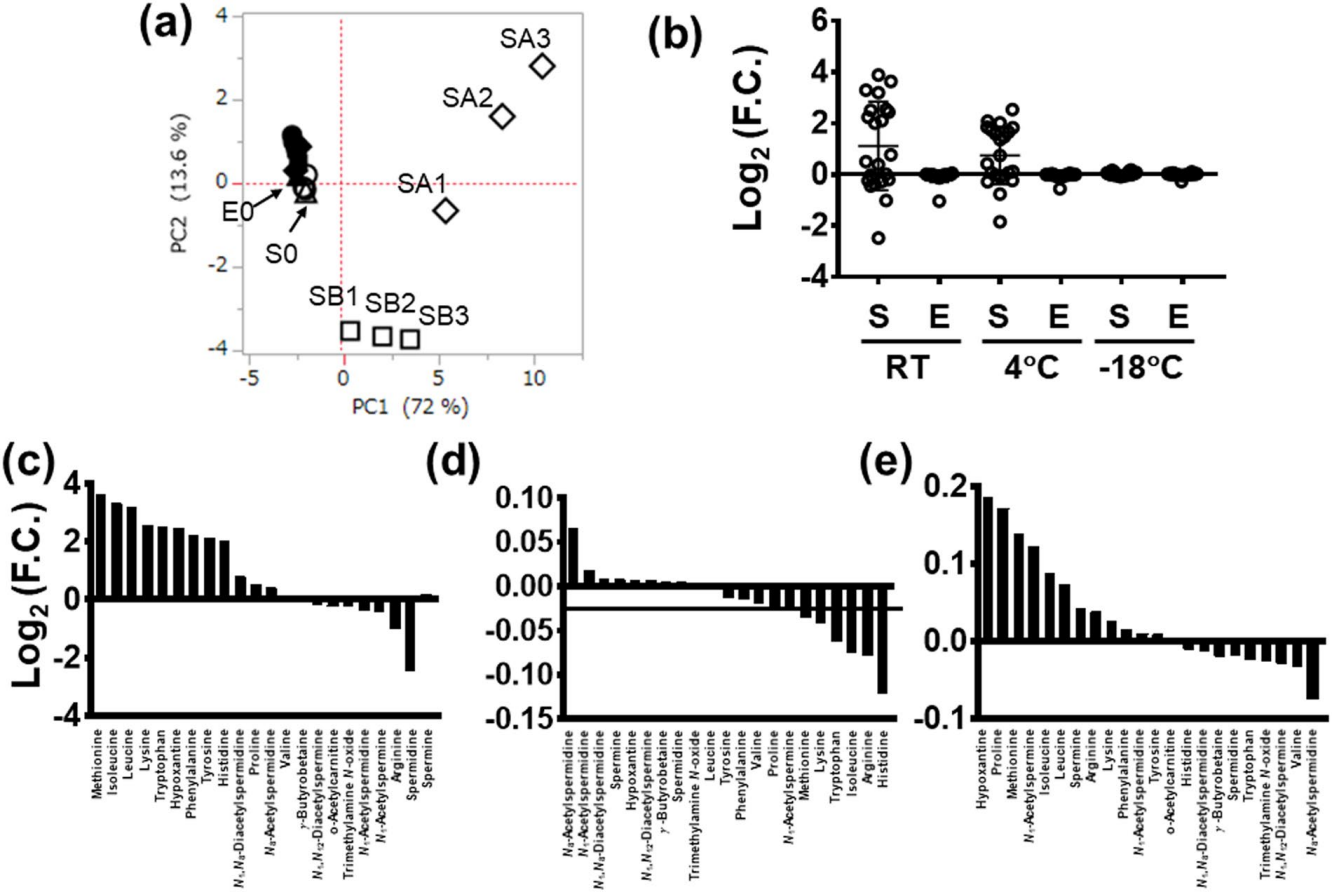
Changes in metabolite concentrations in the long-term storage test. (**a**) Score plots of principal component (PC) analysis. The PC1 and PC2 indicated the first and the second PC. The contributions for PC1 and PC2 were 72.0 and 13.6%, respectively. Filled and open plots indicated samples adding ethanol and internal standards (IS) or not. Labels corresponded to Fig. 1b. (**b**) Log_2_ values of fold change (F.C.) of metabolite concentrations of samples after 8 days. One plot measures one metabolite. S and E indicate samples without and with adding ethanol; RT: room temperature. (**c**) Log_2_ of F.C. in saliva samples without ethanol stored at RT, corresponding to SA3/S0 in Fig. 1b. (**d**) Log_2_ of F.C. in saliva samples with ethanol stored at RT, corresponding to EA3/E0 in Fig. 1b. (**e**) Log_2_ of F.C. in saliva samples without ethanol stored at −18 °C, corresponding to SC3/S0 in Fig. 1b.

A comparison of metabolite concentrations is summarized in Fig. 4. Figure 4a and Fig S1b showed score and loading plots of PCA, respectively. The contribution ratio of PC1 (72%) of this PCA was also higher than that of PC2. The samples treated with ethanol (filled plots) showed a lesser difference in PC1 than those without ethanol addition (open plots). Saliva without ethanol addition and storage at RT and at 4 °C showed more pronounced changes than the samples under the other conditions (Fig. 4b). Saliva without adding ethanol changed significantly under storage at RT, with an increase in amino acids and *N*_1_,*N*_8_-diacetylspermidine and a reduction in all other metabolites (Fig. 4c). The saliva samples treated with ethanol displayed fewer changes (Fig. 4b); all metabolites showed F.C. > 0.9 and F.C. < 1.1 even when stored at RT (Fig. 4d). Without ethanol addition, saliva storage at −18 °C also showed F.C. > 0.9 and <1.1, except for two metabolites: hypoxanthine and proline. The time course of salivary metabolite concentration with and without adding ethanol are depicted in Figs S3 and S4, respectively. Spermine levels increased without adding ethanol and spermidine levels decreased with an increase in amino acid levels. Changes in acetylated polyamines were relatively less pronounced compared to those in amino acids. Upon adding ethanol, these changes were yet lesser pronounced compared to saliva without adding ethanol. The effects of the internal standards (IS) on quantified values were compared and summarized at Table S6.

### Comparison of solvent and drying steps

**Figure 5:**
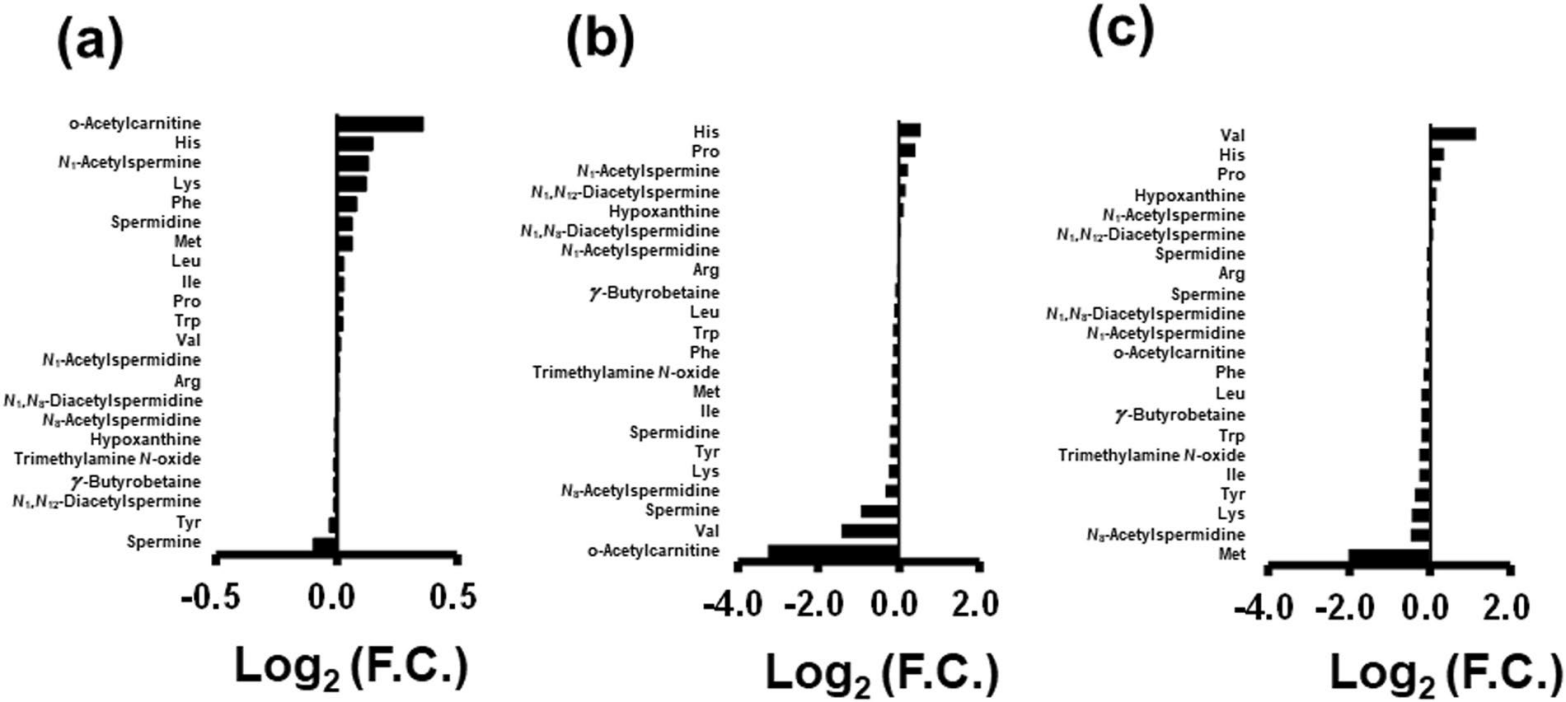
Log_2_ of Fold change (F.C.) of metabolite concentrations comparing saliva samples treated with methanol and a drying process (M1 in Fig. 1c). (**a**) Log_2_ values of F.C. in saliva samples treated with ethanol and dried (E1/M1) (**b**) Log_2_ values of F.C. in saliva samples with adding ethanol but without drying (M2/M1) (**c**) Log_2_ values of F.C. in saliva samples with ethanol adding without drying (E2/M1). The salivary sample with 3.6 μM of STD mixture addition) was used here.

Comparison of metabolite concentrations in saliva containing 0.4 μM (saliva A) and 3.6 μM (saliva B) standard mixture are shown in Figs 5 and S5, respectively, and Table S7. Mean RSD values calculated for 19 metabolites using four replicates of saliva samples were similar when processed with the drying procedure (5% and 4% for saliva A and saliva B for both solvents). Without drying, the values for saliva samples processed with methanol and ethanol were 12% and 6% for saliva A and 6% and 4% for saliva B, with lower values resulting from the data with adding ethanol. The largest RSD values were realized with methanol deproteinization, particularly spermine after drying and valine without drying.

### Salivary markers to distinguish pancreatic cancer

**Figure 6:**
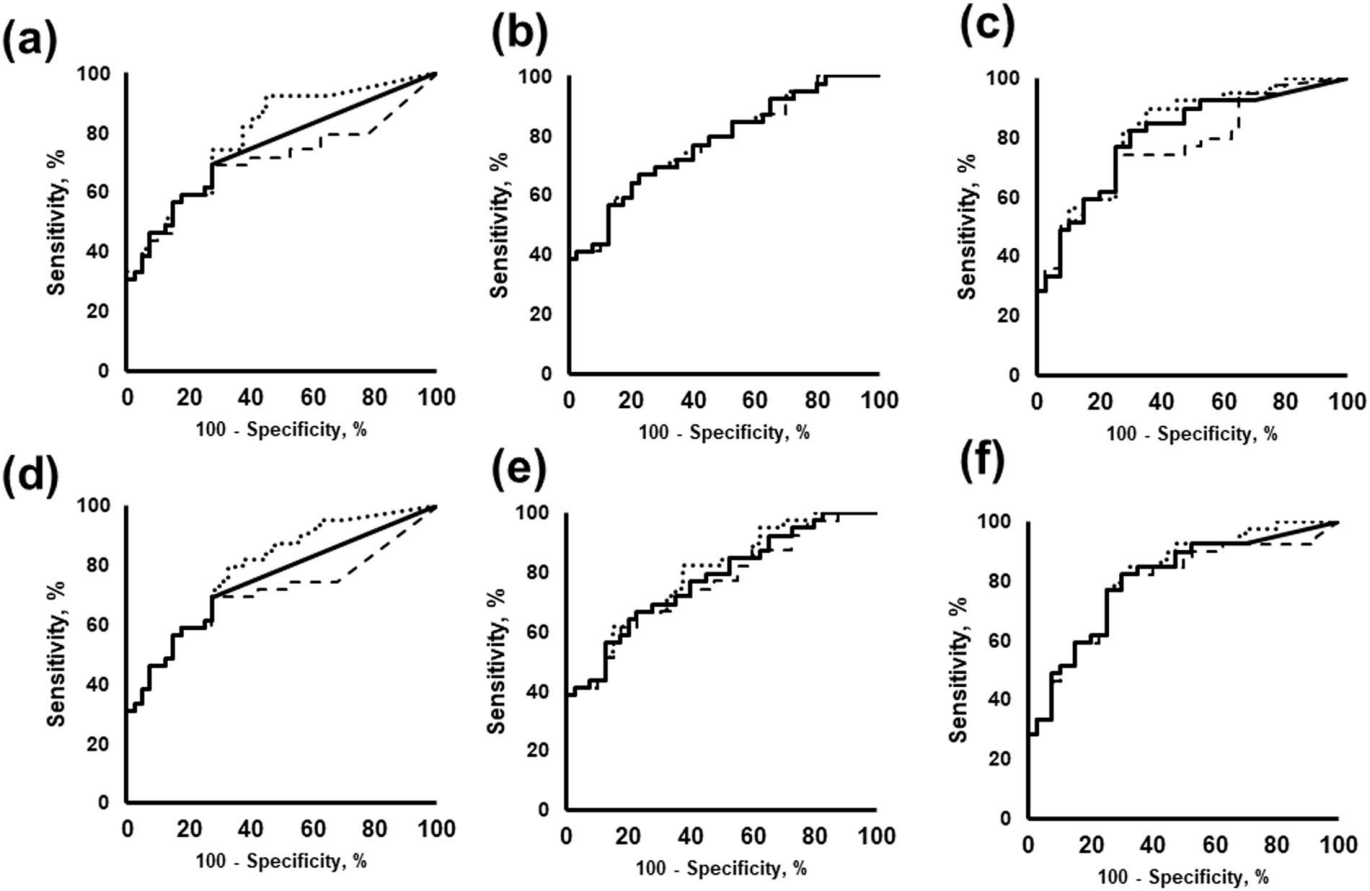
ROC curves of salivary metabolites in discriminating pancreatic cancer (n = 40) from control and chronic pancreatitis (n = 39). (**a**) *N*_1_-acetylspermine, (**b**) *N*_1_-acetylspermidine, and (**c**) spermine in short-term storage test. (**d**) *N*_1_-acetylspermine, (**e**) *N*_1_-acetylspermidine, and (**f**) spermine in the long-term storage test. Solid lines were original data retrieved from the previous study^23^. Dashed lines were generated by adding random noise. In total, 200 curves were generated with random values. ROC curves showing the top 2.5% and 97.5% AUC values are depicted.

We used a computational approach to assess the efficacy of detection of pancreatic cancer based on changes in metabolite concentrations and its discrimination from non-diseased and non-cancer samples. These data were retrieved from our previous study^23^. The largest value of |F.C - 1.0| (i.e. the change in metabolite levels compared to those in fresh samples) during storage was considered a representative change and used as the standard deviation (S.D.) for simulated noise, since several metabolites did not show monotonic trends of increments or reductions. The largest value among the four replicates was chosen for the simulated noise S.D. White noise corresponding to the S.D. was added 200 times for each metabolite, and the AUC values were calculated on the basis of the ROC curves. Variations in AUC values are summarized for short-term and long-term storages (Tables S8 and S9). ROC curves of saliva on ice over short-term storage and saliva under the storage at −18 °C are shown as representative data (Fig. 6**)**.

## Discussion

This study evaluated the effects of various storage conditions on salivary metabolite concentrations after saliva collection. The effects of processing conditions for LC-MS analyses were also evaluated. Metabolomics has been employed frequently to identify biomarkers in salivary samples, especially to diagnose diseases of the oral cavity, including periodontal disease and oral cancer^25^. In addition, biomarkers for systemic disorders, such as cardiovascular disease, have been reported^26^. However, metabolic profiles in biofluid samples need to be considered carefully for analysis. Several studies required a large volume of saliva (3 ml)^27^. A robust and a standardised protocol is hence warranted. To ensure accuracy of the saliva-based analyses, a protocol should be defined and followed to confirm the reproducibility of quantified metabolite concentrations.

This study aimed to evaluate the effect storage and processing conditions on salivary polyamines as potential pancreatic cancer biomarkers. However, amino acids are considered biomarkers for certain other diseases. Our data revealed that changes in salivary metabolite concentrations are less pronounced if saliva is stored on ice or at −18 °C over a short or long term, respectively. At RT, the change in almost every amino acid was larger than those in polyamines. We compared the effect of IS on the RSD of the metabolite concentrations in the samples upon long-tern storage. For example, RSD of spermidine increased from 0.8% to 2.5% using corresponding isotope-labelled compounds to diaminohexan. Changes in metabolite concentrations during the 8 days storage period also included from −1.0 to −1.22 (log_2_ of fold change), using these internal standards. These data indicate that concentration reproducibility of amino acids would be improved, e.g. via isotope-labelled amino acids for more reliable calculations. Since the constant ratio between isotope-labelled and endogenous metabolite concentrations during the storage is expected, the addition of isotope-labelled reagents to saliva samples upon the collection of samples would realize more accurate quantifications.

Furthermore, the present data revealed that the solvent (methanol and ethanol) exerted a minimal effect on metabolite concentrations. In contrast, drying procedures resulted in a significant difference in salivary metabolite concentrations. In particular, the concentration of two amino acids (valine and methionine) was significantly altered by the drying process, which is attributable to the relatively small signal/noise ratio of these metabolites and consequently the skewed peak shapes in particular, compared to that of the other amino acids (Table S7 and Fig. S6). The drying process should be employed uniformly, rather than as an alternative to obtain reproducible profiles. The salivary polyamines retain high discriminative ability for pancreatic cancers even in the noise added data considering the change in marker concentrations during storage.

A similar study investigated the concentration reproducibility of four metabolites, including choline as a biomarker for oral cancer, under various storage conditions^20^. Although we focused on only a portion of available polyamines, all observable metabolites should be evaluated if metabolites are considered potential biomarkers. We previously evaluated the effects of environmental factors^28^ and fasting^18^ on metabolite concentrations. All studies were conducted to detect absolute concentrations. For urinary metabolite biomarkers, determination of creatinine concentration is a common method to normalize the data and eliminate individual variations. Potential metabolites for normalisation against other metabolite concentrations should be explored for saliva.

This study has the following limitations. Herein, we focused on only the effect of storage conditions after sample collection. Toward the establishment of standard operation procedure (SOP) for the use of salivary polyamines as biomarkers, the following aspect also should have been investigated: (1) effect of diary environments, e.g. polyamine intake, and (2) sample collection protocol, e.g. the fasting time before sample collection^18^. Polyamine intake reportedly influenced the risk of colon cancer^29^ and more directly, urinary metabolites^30^, which also potentially influence the salivary polyamines. Sample processing, e.g. the use of serum and plasma obtained from blood samples, also influences the reproducibility of metabolite quantification^31^. The scope of the current study is limited to only unstimulated whole saliva; therefore, a similar study is required for the use of stimulated saliva^32^. These effects should be minimized to accurately evaluate the discriminative ability of salivary polyamines as biomarkers. Regarding the analytical aspects, measurements at multiple time points with various dilutions would be more time-consuming and costly. The reproducibility of quantification methods depended on the selection of internal standards. To allow for high-throughput analyses, single sample processing protocols should be established.

In conclusion, upon short-term storage tests, the saliva samples after collection should be treated with on ice. If saliva samples have to be treated at RT, preservation time should not exceed 30 min. Regarding long-term storage tests, the samples should be stored at −18 °C. If the saliva samples are stored at RT or 4 °C, ethanol and internal standard should be added to the salivary samples. For processing conditions, the use of ethanol and methanol and the use of drying process should not be changed to minimize their effects on the quantified values of salivary polyamines.

## Electronic supplementary material

Supplementary Information

## Data Availability

The datasets generated during and/or analysed during the current study are available from the corresponding author on reasonable request.
