# Supplementary material for: Effect of storage conditions on salivary polyamines quantified via liquid chromatography-mass spectrometry
Source: Sci Rep. 2018 Aug 13;8:12075. doi: 10.1038/s41598-018-30482-x (PMC6089938; doi:10.1038/s41598-018-30482-x)
Supplement: Supplementary file 1 — Supplementary Information [file 41598_2018_30482_MOESM1_ESM.pdf]

Supplementary Information for

Effect of storage conditions on salivary polyamines quantified via liquid chromatography mass spectrometry

Atsumi Tomita[1], Masayo Mori[2], Kana Hiwatari[2], Eri Yamaguchi[1], Takao Itoi[3], Makoto Sunamura[4], Tomoyoshi Soga[2], Masaru Tomita[2], Masahiro Sugimoto[12*]

[1]Health Promotion and Preemptive Medicine, Research and Development Center for Minimally Invasive Therapies, Tokyo Medical University, Shinjuku, Tokyo, 160-8402, Japan
[2]Institute for Advanced Biosciences, Keio University, Tsuruoka, Yamagata 997-0052, Japan
[3]Division of Gastroenterology and Hepatology, Tokyo Medical University, Shinjuku, Tokyo 160-0023, Japan
[4]Fourth Department of Surgery, Tokyo Medical University Hachioji Medical Center, Hachioji, Tokyo 190-0998, Japan

***Correspondence:** Masahiro Sugimoto
Minimally Invasive Therapies, Tokyo Medical University, Shinjuku, Tokyo, 160-8402, Japan, E-mail: mshrsgmt@tokyo-med.ac.jp or mshrsgmt@gmail.com; Tel.: +81-3-3351-6141

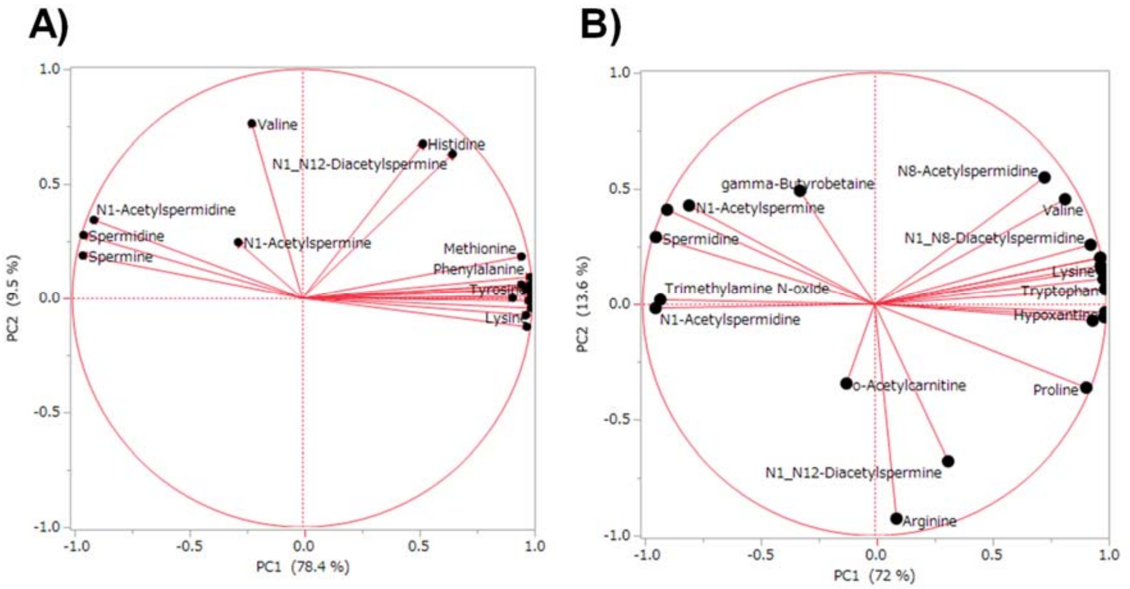

Figure S1 Loading plots of principal component (PC) analyses. The PC1 and PC2 indicated the first and the second PC. Short-term **A)** and Long-term storage tests **B)**.

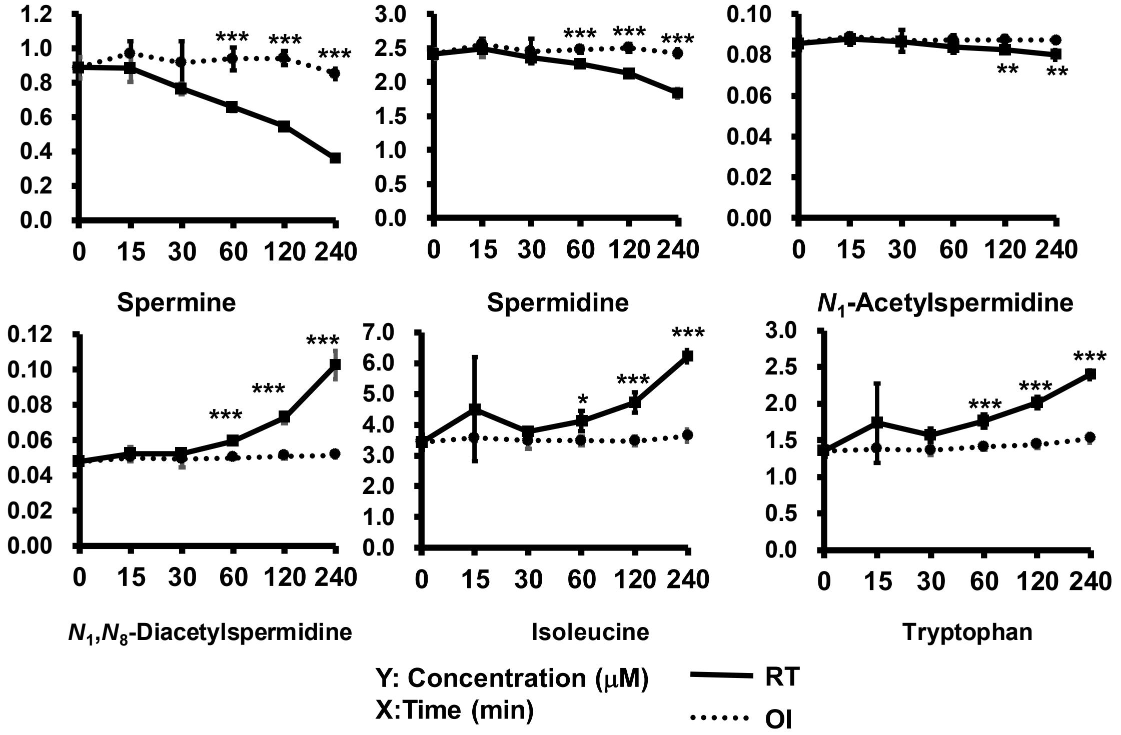

Figure S2 Representative time-courses of mean metabolite concentrations during short-term storage tests. X and Y axes indicate storage time (min) and concentration (μM). S.D (n=4) is indicated with error bars. Room temperature (RT) and on ice (OI) indicate 22 ºC and on ice. **P* < 0.05, ***P* < 0.01 and *** *P* < 0.001 (Student's t-*t*est, both tail).

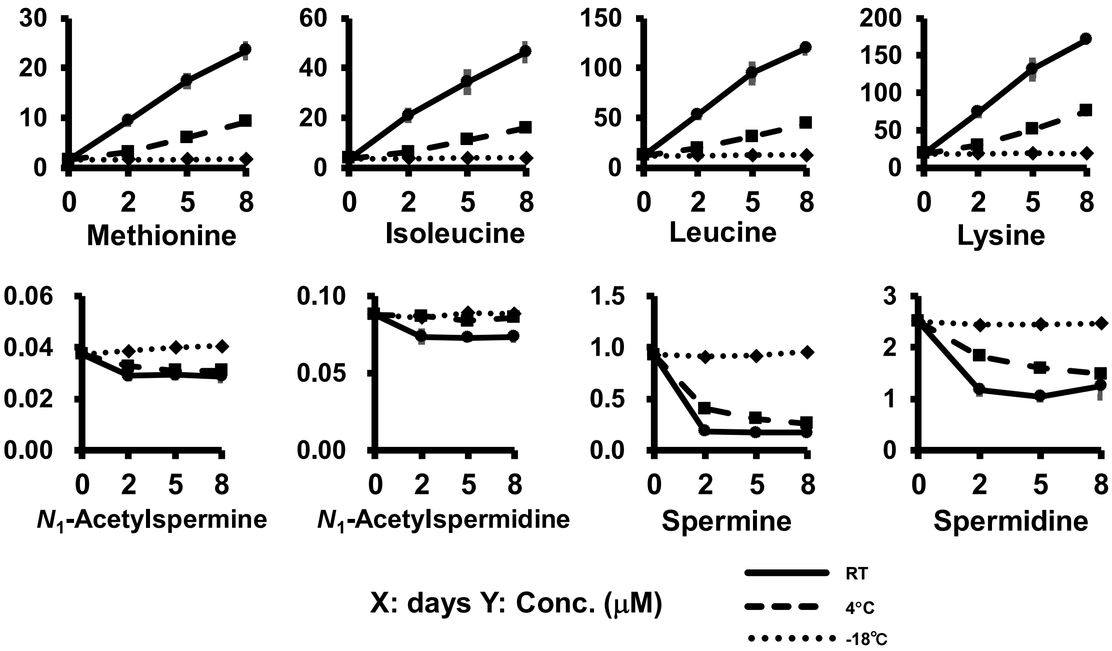

Figure S3 Representative time-courses of mean metabolite concentrations in saliva during long-term storage tests. X and Y axes indicate storage time (days) and concentration (μM). S.D (n=4) is indicated with error bars. *$P < 0.05$, **$P < 0.01$ and *** $P < 0.001$ (Student's *t*-test, both tail).

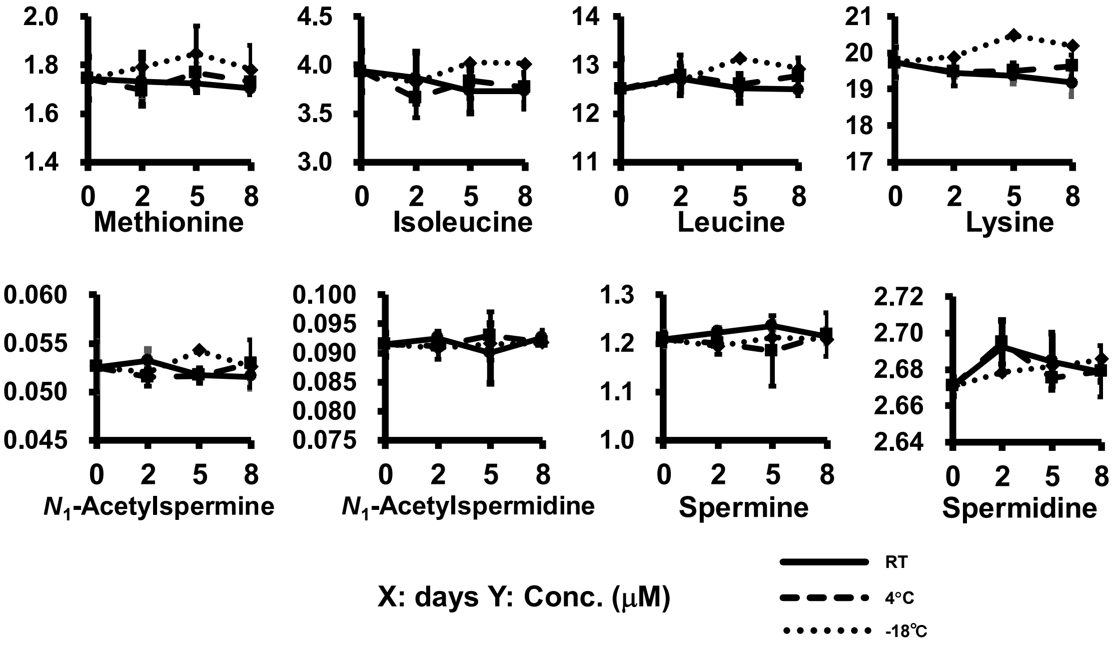

Figure S4. Representative time-courses of mean metabolite concentrations in saliva with ethanol addition and IS addition during long-term storage tests. X and Y axes indicate storage time (days) and concentration (μM). S.D (n=4) is indicated with error bars. *$P < 0.05$, **$P < 0.01$ and *** $P < 0.001$ (Student's *t*-test, both tail).

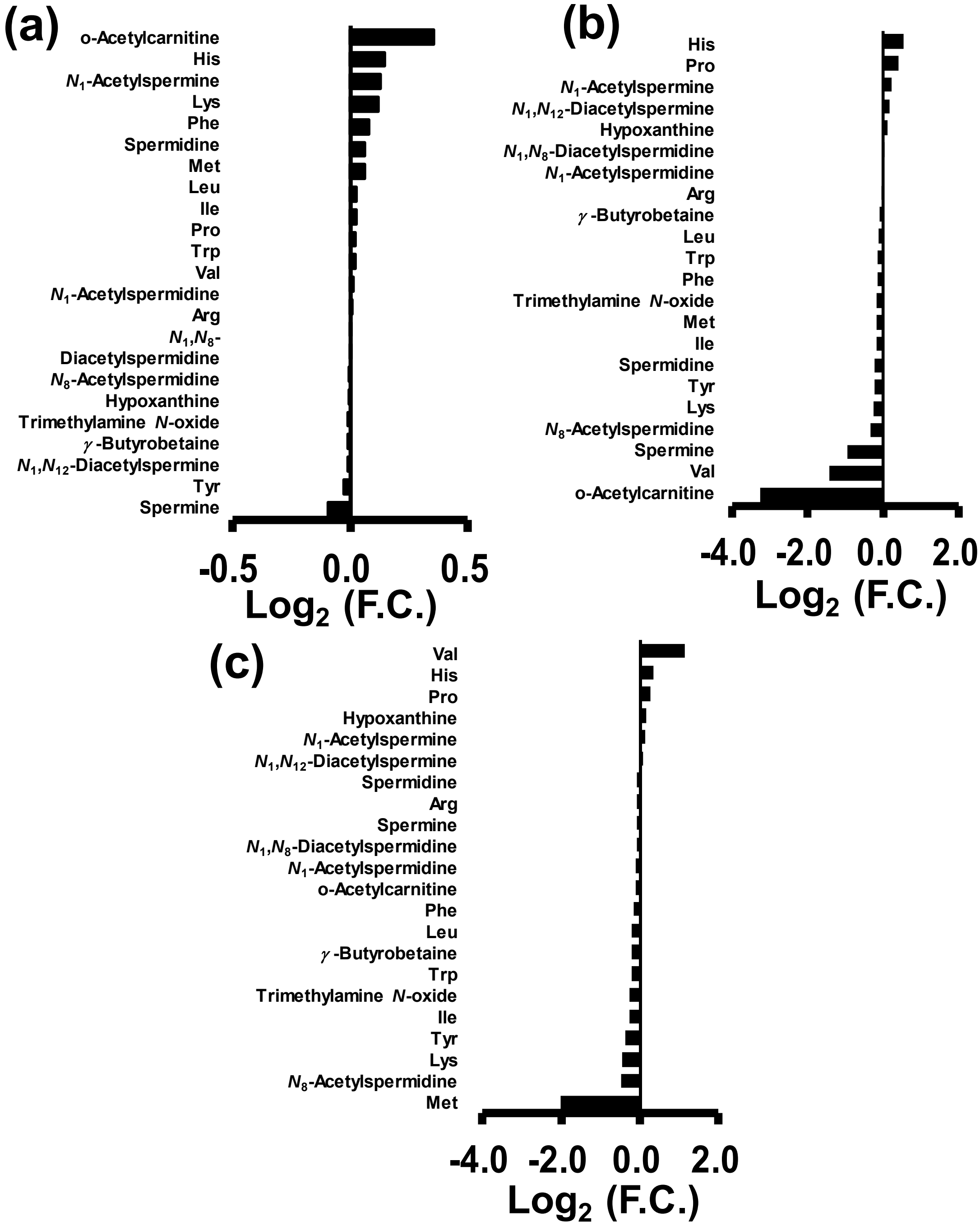

Figure S5 Fold change (F.C.) of metabolite concentrations comparing saliva spiked with 0.4 μM of STD mixture, with methanol adding and drying step (M1 in **Fig 1c**). (**a**) F.C. of samples with ethanol adding and drying step (E1/M1) (**b**) F.C. of samples with methanol adding but no drying (M2/M1) (**c**) F.C. of samples with ethanol adding but no drying (E2/M1).

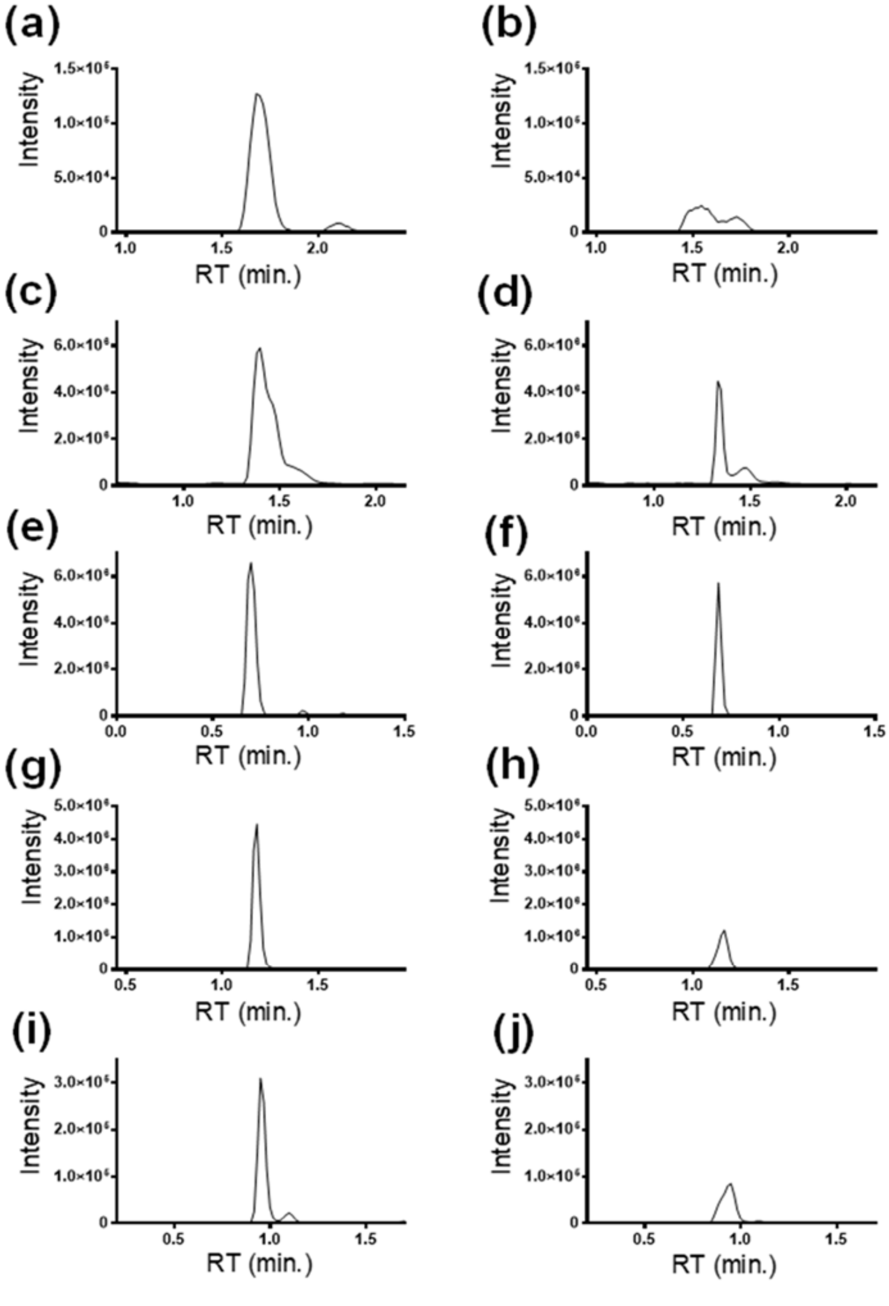

Figure S6. Extracted ion chromatography of amino acids. **(a) and (b)** Methionine (150.0583 *m/z* and 1.68 min). **(c) and (d)** Valine (118.0863 *m/z* and 1.62 min). **(e) and (f)** Proline (116.0706 *m/z* and 0.70 min). **(g) and (h)** EIC Arginine (175.1190 *m/z* and 1.18 min). **(i) and (j)** Histidine (156.0768 *m/z* and 0.95 min). **(a), (c), (e), (g) and (i)** Saliva samples with ethanol adding and a drying step. **(b), (d), (f), (h) and (j)** Saliva samples with ethanol adding but without drying step.

Table S1. Metabolite concentration listed in Human Metabolome Database [1]

HMDB ID<br>PMID	Metabolite	Concentration range (μM)	Sex	Reference
HMDB0000517	Arg	5.2 +/- 1.7	male	Nakamura et al.(2010) [2]
HMDB0000177	His	6.02 +/- 4.39	female	Sugimoto et al. (2013) [3]
HMDB0000157	Hypoxanthine	1.57 +/- 1.14	both	Sugimoto et al. (2013) [3]
HMDB0000172	Ile	6.60 +/- 9.47	male	Sugimoto et al. (2013) [3]
HMDB0000687	Leu	2.79 +/- 1.21	female	Sugimoto et al. (2013) [3]
HMDB0000182	Lys	19.2 +/- 12.8	female	Sugimoto et al. (2013) [3]
HMDB0000696	Met	2.88 +/- 5.55	male	Sugimoto et al. (2013) [3]
HMDB0002172	N_1,N_{12}-Diacetylspermine	-		
HMDB0041947	N_1,N_8-Diacetylspermidine	-		
HMDB0001276	N_1-Acetylspermidine	-		
HMDB0001186	N_1-Acetylspermine	-		
HMDB0002189	N_8-Acetylspermidine	0.15 +/- 0.0787	female	Sugimoto et al. (2013) [3]
HMDB0000159	Phe	5.55 +/- 4.30	female	Sugimoto et al. (2013) [3]
HMDB0000162	Pro	18.8 +/- 13.5	female	Sugimoto et al. (2013) [3]
HMDB0001257	Spermidine	0.402 +/- 0.320	female	Sugimoto et al. (2013) [3]
HMDB0001256	Spermine	0.242 +/- 0.565	female	Sugimoto et al. (2013) [3]
HMDB0000929	Trp	0.46 +/- 0.31	female	Tsuruoka et al(2013) [4]
HMDB0000158	Tyr	11.1 +/- 7.65	female	Sugimoto et al. (2013) [3]
HMDB0000883	Val	3.63 +/- 1.66	both	Sugimoto et al. (2013) [3]

The concentrations were retrieved from the healthy subjects' data registered.

References

1 Wishart, D. S. *et al.* HMDB 4.0: the human metabolome database for 2018. *Nucleic Acids Res* **46**, D608-D617, doi:10.1093/nar/gkx1089 (2018).

2 Nakamura, Y. *et al.* Diurnal changes in salivary amino acid concentrations. *In Vivo* **24**, 837-842 (2010).

3 Sugimoto, M. *et al.* Physiological and environmental parameters associated with mass spectrometry-based salivary metabolomic profiles. *Metabolomics : Official journal of the Metabolomic Society* **9**, 454-463 (2013).

4 Tsuruoka, M. *et al.* Capillary electrophoresis-mass spectrometry-based metabolome analysis of serum and saliva from neurodegenerative dementia patients. *Electrophoresis* **34**, 2865-2872, doi:10.1002/elps.201300019 (2013).

Table S2. LC gradients for polyamine analysis

Time	Mobile phase	
(min)	A (%)	B (%)
0.0	99	1
0.6	99	1
0.8	58	42
1.8	58	42
2.3	50	50
3.0	50	50
4.0	5	95
5.0 (QQQ-MS) / 6.0 (TOF-MS)	5	95

Table S3. MRM scans for polyamines and amino acids

Compound	Q1 Precursor ion (*m/z*)	Q3 Product ion (*m/z*)	Fragmentor (v)	CE (v)	CAV (v)	RT (min)
1,6-Diaminohexane	117.1	100.1	70	9	4	2.66
Spermidine-d_8	154.2	32.1	95	41	4	3.43
*N*1-Acetylspermidine-d_6	194.2	106.0	100	17	4	2.92
Spermine-d_8	211.3	120.1	100	21	4	3.96
Pro-$^{13}C_5$,^{15}N	122.1	75.1	85	17	4	0.542
Lys-$^{13}C_6$,$^{15}N_2$	155.1	90.1	80	17	4	0.768
Arg-$^{13}C_6$,$^{15}N_4$	185.1	75.2	110	29	4	0.930
Leu-d_3	135.1	30.2	80	17	4	3.03
Phe-d_5	171.1	125.1	85	9	4	3.20
Met-$^{13}C_5$,^{15}N	156.1	109.0	75	9	4	1.53
Trp-$^{13}C_{11}$,$^{15}N_2$	218.1	200.1	85	9	4	3.54
*N*1,*N*8-Diacetylspermidine-d_6	236.2	103.1	125	17	4	2.78
*N*1,*N*12-Diacetylspermine-d_6	293.3	103.1	120	25	4	3.26
Pro	116.1	70.1	80	13	4	0.542
Val	118.1	72.1	65	9	4	1.41
Ile	132.1	86.1	70	9	4	2.94
Leu	132.1	86.1	80	9	4	3.03
Hypoxanthine	137.0	110.0	125	21	4	0.642
Spermidine	146.2	72.1	95	13	4	3.43
Lys	147.1	84.1	85	17	4	0.768
Met	150.1	104.0	80	9	4	1.53
His	156.1	110.0	90	13	4	0.698
Phe	166.1	120.0	75	9	4	3.20
Arg	175.1	70.1	110	25	4	0.930
Tyr	182.1	91.1	85	29	4	2.53
*N*1-Acetylspermidine	188.2	100.0	105	17	4	2.93
*N*8-Acetylspermidine	188.2	114.0	115	17	4	3.06
Spermine	203.2	112.0	100	17	4	3.96
Trp	205.1	188.0	85	9	4	3.54
*N*1,*N*8-Diacetylspermidine	230.2	100.0	120	17	4	2.78
*N*1-Acetylspermine	245.2	100.0	110	21	4	3.64
*N*1,*N*12-Diacetylspermine	287.2	100.0	125	25	4	3.26
γ-Butylbetaine	146.1	45.1	90	29	4	0.914
Trimethylamine *N*-oxide	76.1	58.1	60	21	4	0.818
o-Acetylcarnitine	204.1	85.1	105	21	4	2.27
N-Acetylptrecine	131.1	114.0	85	9	4	1.41
Pipecolate	130.1	84.1	85	17	4	1.11
Adenosine	268.1	136.0	110	13	4	2.57
Creatinine	114.1	44.2	105	17	4	0.806

CE; collision energy, CAV; cell accelerator voltage, Q1; first quadrupole, Q3; last quadrupole

Table S4. Recovery and RSD of salivary polyamines and amino acids

Metabolite	Saliva A[1]		Saliva B[1]	
	Recovery (%)	RSD[2] (%)	Recovery (%)	RSD[2] (%)
1,6-Diaminohexane	97.6	2.1	106	2.1
Arg-$^{13}C_6$,$^{15}N_4$	71.9	2.9	67.9	2.9
Lys-$^{13}C_6$,$^{15}N_2$	60.5	2.6	54.5	4.4
Met-$^{13}C_5$,^{15}N	90.6	2.5	87.5	2.8
Pro-$^{13}C_5$,^{15}N	31.5	3.2	28.2	4.1
Trp-$^{13}C_{11}$,$^{15}N_2$	101	2.5	103	2.4
Leu-d_3	95.9	2.1	98.5	2.4
Phe-d_5	94.2	3.1	97.0	1.7
N_1-Acetylspermidine-d_6	116	2.6	118	1.1
Spermidine-d_8	79.4	1.8	102	3.4
Spermine-d_8	58.9	5.2	102	4.4

[1]A and B indicate individual patient samples. [2]RSD indicates relative standard deviation calculated by peak area.

Table S5 Calibration curves of polyamines and amino acids (X and Y are concentration and relative area, respectively)

Metabolites	Arg	
Calibration curves	Linear	Log_{10}
	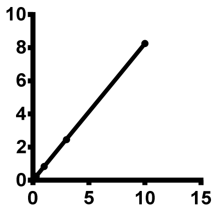	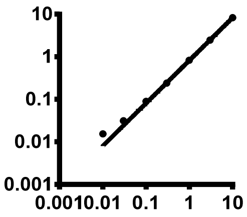
Equation	Y = 0.8252 × X + 0.0003248	
Goodness fit	1.0000	
RSD (%)	0.5	
Range	0.01 – 10 μM	
Metabolites	His	
Calibration curves	Linear	Log_{10}
	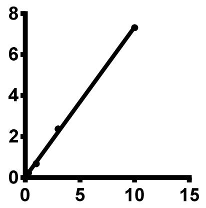	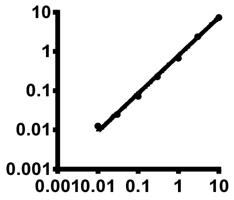
Equation	Y = 0.7341 × X + 0.01328	
Goodness fit	0.9995	
RSD (%)	0.5	
Range	0.01 – 10 μM	
Metabolites	Hypoxanthine	
Calibration curves	Linear	Log_{10}
	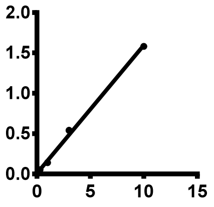	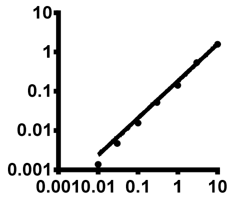
Equation	Y = 0.159 × X + 0.005697	
Goodness fit	0.9987	
RSD (%)	0.5	
Range	0.01 – 10 μM	

Table S5 (Continued)

Metabolites	Ile	
Calibration curves	Linear	Log_{10}
	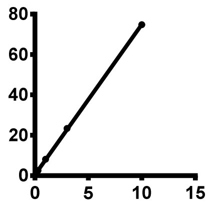	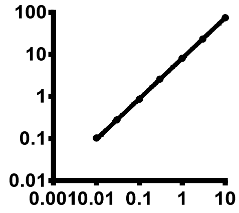
Equation	Y = 7.463 × X + 0.3344	
Goodness fit	1.0000	
RSD (%)	0.5	
Range	0.01 – 10 μM	
Metabolites	Leu	
Calibration curves	Linear	Log_{10}
	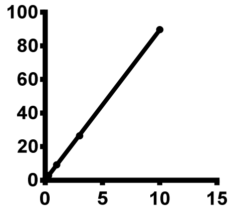	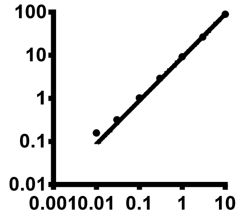
Equation	Y = 8.94 × X + 0.08192	
Goodness fit	0.9999	
RSD (%)	0.5	
Range	0.01 – 10 μM	
Metabolites	Lys	
Calibration curves	Linear	Log_{10}
	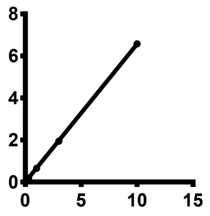	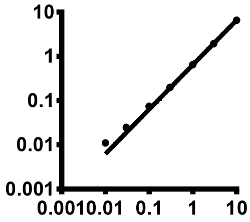
Equation	Y = 0.6567 × X – 0.0008836	
Goodness fit	1.0000	
RSD (%)	0.5	
Range	0.01 – 10 μM	

Table S5 (Continued)

Metabolites	Met	
Calibration curves	Linear	Log_{10}
	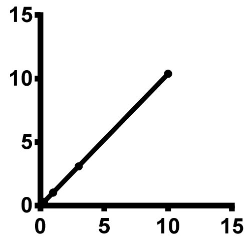	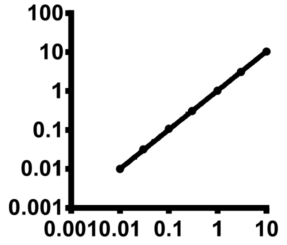
Equation	Y = 1.037 × X – 0.003882	
Goodness fit	1.0000	
RSD (%)	0.5	
Range	0.01 – 10 μM	
Metabolites	N_1,N_{12}-Diacetylspermine	
Calibration curves	Linear	Log_{10}
	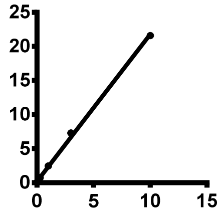	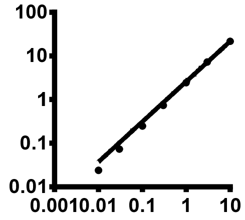
Equation	Y = 2.163 × X + 0.1703	
Goodness fit	0.9998	
RSD (%)	0.5	
Range	0.01 – 10 μM	
Metabolites	N_1,N_8-Diacetylspermidine	
Calibration curves	Linear	Log_{10}
	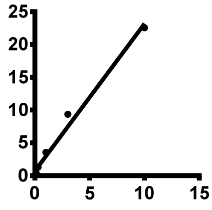	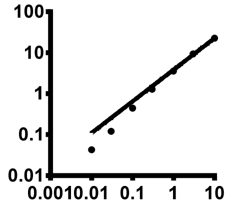
Equation	Y = 2.249 × X + 0.7002	
Goodness fit	0.9991	
RSD (%)	0.5	
Range	0.01 – 10 μM	

Table S5 (Continued)

Metabolites	*N*1-Acetylspermidine	
Calibration curves	Linear	Log_{10}
	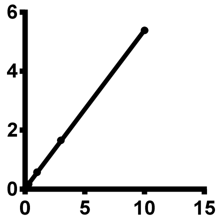	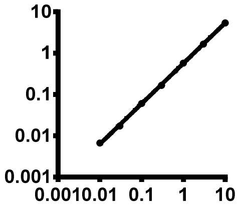
Equation	Y = 0.5384 × X + 0.01357	
Goodness fit	1.0000	
RSD (%)	0.5	
Range	0.01 – 10 μM	
Metabolites	*N*1-Acetylspermine	
Calibration curves	Linear	Log_{10}
	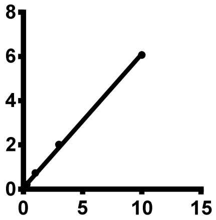	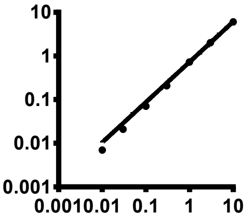
Equation	Y = 0.6066 × X + 0.05151	
Goodness fit	0.9999	
RSD (%)	0.5	
Range	0.01 – 10 μM	
Metabolites	*N*8-Acetylspermidine	
Calibration curves	Linear	Log_{10}
	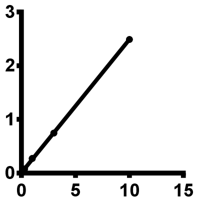	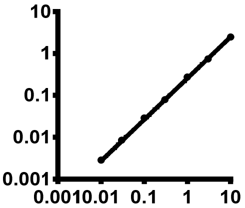
Equation	Y = 0.2483 × X + 0.006107	
Goodness fit	0.9999	
RSD (%)	0.5	
Range	0.01 – 10 μM	

Table S5 (Continued)

Metabolites	Phe	
Calibration curves	Linear	Log_{10}
	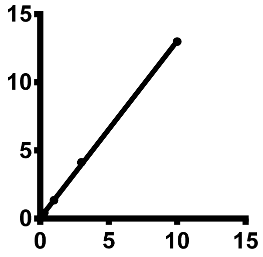	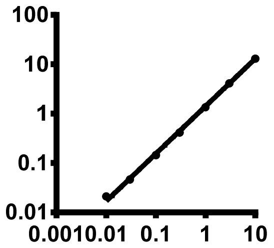
Equation	Y = 1.299 × X + 0.04894	
Goodness fit	0.9999	
RSD (%)	0.5	
Range	0.01 – 10 μM	
Metabolites	Pro	
Calibration curves	Linear	Log_{10}
	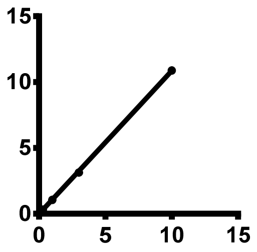	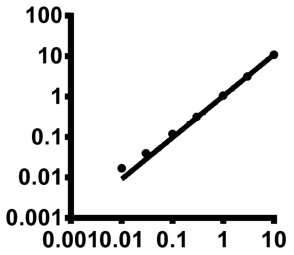
Equation	Y = 1.087 × X – 0.01874	
Goodness fit	1.0000	
RSD (%)	0.5	
Range	0.01 – 10 μM	
Metabolites	Spermidine	
Calibration curves	Linear	Log_{10}
	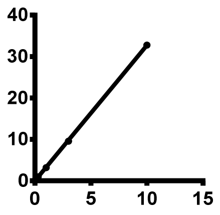	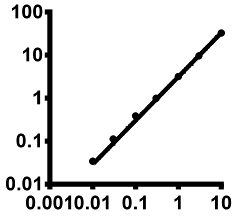
Equation	Y = 3.276 × X – 0.03338	
Goodness fit	1.0000	
RSD (%)	0.5	
Range	0.01 – 10 μM	

Table S5 (Continued)

Metabolites	Spermine	
Calibration curves	Linear	Log_{10}
	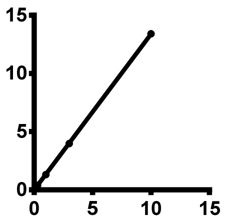	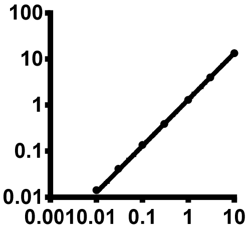
Equation	Y = 1.34 × X – 0.01188	
Goodness fit	1.0000	
RSD (%)	0.5	
Range	0.01 – 10 μM	
Metabolites	Trp	
Calibration curves	Linear	Log_{10}
	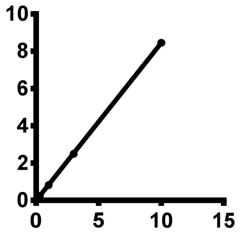	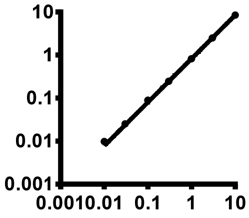
Equation	Y = 0.8455 × X – 0.008884	
Goodness fit	1.0000	
RSD (%)	0.5	
Range	0.01 – 10 μM	
Metabolites	Tyr	
Calibration curves	Linear	Log_{10}
	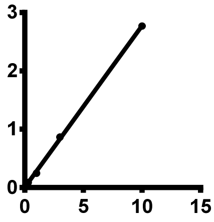	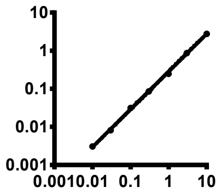
Equation	Y = 0.2776 × X – 0.0001932	
Goodness fit	0.9997	
RSD (%)	0.5	
Range	0.01 – 10 μM	

Table S5 (Continued)

Metabolites	Val	
Calibration curves	Linear	Log_{10}
	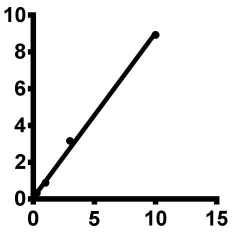	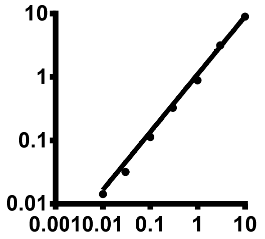
Equation	Y = 0.8965 × X + 0.07437	
Goodness fit	0.9988	
RSD (%)	0.5	
Range	0.01 – 10 μM	

X and Y axes of calibration curves indicate concentration (μM) and relative area, i.e. area of a metabolite peak/area of an internal standard peak (no unit). Double–log_{10}–scale and linear showed log_{10} and linear values, respectively, of both X and Y axes. Ordinary least squares was used for linear regressions. All figures included 7 points data. RSD was calculated by the relative area in the 1μM of a standard mixture (inter-day variance, n = 5). Goodness fit provides R^2 values.

Table S6. The reproducibility of quantified metabolites using various internal standards (IS) in long-term storage test

Metabolites	IS1		IS2		IS3		IS4		IS5		IS6		IS7		IS8	
	RSD	F.C.	RSD	F.C.	RSD	F.C.	RSD	F.C.	RSD	F.C.	RSD	F.C.	RSD	F.C.	RSD	F.C.
Arg	**2.1**	**-0.439**	3.7	1.53	1.9	-0.346	1.9	-0.565	1.1	-0.496	2.1	-0.196	4.7	-0.227	2.8	0.323
His	**1.6**	**2.12**	4.5	4.07	1.6	2.21	2.4	1.99	1.5	2.06	1.2	2.36	3.6	2.33	1.9	2.87
Hypoxanthine	5.1	0.563	**1.2**	**2.52**	4.4	0.658	2.8	0.438	3.3	0.507	5.1	0.810	7.9	0.779	5.9	1.32
Ile	**3.7**	**3.65**	3.2	5.60	3.3	3.74	2.2	3.52	2.6	3.59	4.0	3.90	6.7	3.86	4.7	4.41
Leu	**2.9**	**3.31**	4.3	5.26	2.3	3.40	2.7	3.18	2.5	3.25	2.5	3.55	4.5	3.52	3.4	4.07
Lys	**1.0**	**3.20**	4.6	5.15	0.83	3.30	2.3	3.08	1.5	3.15	0.86	3.45	3.5	3.42	1.7	3.96
Met	**6.5**	**3.89**	3.1	5.84	5.7	3.99	4.8	3.77	5.3	3.84	6.8	4.14	9.4	4.11	7.5	4.65
N_1,N_{12}-Diacetylspermine	1.6	-0.109	3.9	1.84	**1.0**	**-0.014**	1.5	-0.233	0.92	-0.164	1.5	0.137	4.3	0.107	2.4	0.651
N_1,N_8-Diacetylspermidine	2.9	2.13	2.7	4.08	2.4	2.23	**0.57**	**2.01**	1.3	2.08	2.9	2.38	5.7	2.35	3.8	2.89
N_1-Acetylspermidine	1.8	-0.207	3.6	1.74	1.5	-0.112	1.4	-0.330	**0.41**	**-0.262**	1.9	0.040	4.6	0.00934	2.6	0.553
N_1-Acetylspermine	1.1	-0.620	4.6	1.34	1.3	-0.526	2.4	-0.744	1.3	-0.675	**0.79**	**-0.373**	3.5	-0.404	1.6	0.140
N_8-Acetylspermidine	1.3	0.567	4.1	2.51	1.1	0.662	1.8	0.445	**0.79**	**0.514**	1.3	0.816	4.1	0.785	2.1	1.33
Phe	**3.2**	**2.45**	2.5	4.40	2.5	2.54	1.1	2.32	1.6	2.39	3.2	2.69	5.9	2.66	4.0	3.21
Pro	**2.8**	**0.782**	4.6	2.73	2.6	0.877	2.4	0.659	2.3	0.727	2.8	1.03	4.9	0.998	3.4	1.54
Spermidine	2.5	-1.22	7.3	0.718	3.0	-1.13	4.9	-1.34	4.2	-1.28	2.4	-0.974	**0.8**	**-1.00**	1.9	-0.466
Spermine	1.7	-3.23	6.5	-1.28	2.5	-3.13	4.2	-3.35	3.3	-3.28	1.6	-2.98	1.7	-3.01	**0.95**	**-2.47**
Trp	**5.1**	**2.55**	1.7	4.50	4.6	2.65	3.0	2.43	3.4	2.50	5.2	2.80	8.0	2.77	6.0	3.31
Tyr	**2.9**	**2.24**	3.2	4.19	2.4	2.34	1.8	2.12	1.5	2.19	2.7	2.49	5.4	2.46	3.6	3.00
Val	**2.6**	**0.399**	4.5	2.35	2.3	0.493	3.0	0.275	2.6	0.344	2.7	0.645	4.9	0.614	3.1	1.16
γ-Butyrobetaine	**2.8**	**0.024**	2.8	1.98	2.2	0.118	1.1	-0.100	1.4	-0.031	2.9	0.270	5.6	0.240	3.7	0.784
Trimethylamine *N*-oxide	**0.70**	**-0.231**	4.8	1.72	0.89	-0.137	2.4	-0.355	1.6	-0.286	1.0	0.015	3.3	-0.016	1.6	0.530
o-Acetylcarnitine	**4.9**	**-0.188**	7.8	1.77	5.1	-0.094	5.5	-0.315	5.4	-0.245	4.6	0.055	4.9	0.024	4.6	0.573

RSD was calculated by the relative area in the 1μM of standard mixture. Log_2 of F.C. was calculated by the relative area of the peaks in saliva samples after 8 days / those of initial samples. For internal standards, diaminohexane (IS1), hypoxanthine-$^{13}C_2$,^{15}N (IS2), d_6-N_1,N_{12}-diacetylspermine (IS3), d_6-N_1,N_8-diacetylspermidine (IS4), d_6-N_1-Acetylspermidine (IS5), d_3-N_1-Acetylspermine (IS6), d_8-Spermidine (IS7), and d_8-Spermine (IS8). Data in bold indicated the pairs of a metabolite and a corresponding internal standard used for all of the other experiments.

Table S7. Effect of processing condition of metabolite concentrations and S/N

Metabolite	With drying								Without drying							
	Methanol				Ethanol				Methanol				Ethanol			
	Saliva A[1]		Saliva B[1]		Saliva A[1]		Saliva B[1]		Saliva A[1]		Saliva B[1]		Saliva A[1]		Saliva B[1]	
	Ave ± SD	S/N	Ave ± SD	S/N	Ave ± SD	S/N	Ave ± SD	S/N	Ave ± SD	S/N	Ave ± SD	S/N	Ave ± SD	S/N	Ave ± SD	S/N
Arg	35.9 ± 0.797	8.70×10^{3}	34.0 ± 0.672	1.75×10^{4}	36.7 ± 0.934	7.42×10^{3}	34.2 ± 0.590	9.21×10^{3}	35.2 ± 1.05	1.85×10^{3}	33.1 ± 1.44	2.48×10^{3}	34.7 ± 1.68	1.67×10^{3}	32.0 ± 0.566	1.54×10^{3}
His	7.29 ± 0.226	1.27×10^{2}	6.28 ± 0.494	4.04×10^{2}	6.32 ± 0.425	2.03×10^{2}	6.94 ± 0.593	4.69×10^{2}	8.16 ± 0.763	1.52×10^{2}	9.07 ± 0.197	1.02×10^{2}	8.20 ± 0.745	2.00×10^{2}	7.99 ± 0.701	3.42×10
Hypoxanthine	3.23 ± 0.0314	6.20×10^{2}	6.92 ± 0.215	6.40×10^{2}	3.22 ± 0.0485	8.13×10^{2}	6.90 ± 0.0926	6.03×10^{2}	3.45 ± 0.221	1.97×10^{2}	7.56 ± 0.279	2.54×10^{2}	3.54 ± 0.209	4.61×10^{2}	7.78 ± 0.248	6.25×10^{2}
Ile	4.40 ± 0.387	2.97×10^{2}	7.46 ± 0.189	6.09×10^{2}	4.48 ± 0.290	3.67×10^{2}	7.59 ± 0.109	8.35×10^{2}	3.90 ± 0.284	1.42×10^{2}	6.60 ± 0.427	2.38×10^{2}	3.70 ± 0.243	1.01×10^{2}	6.19 ± 0.141	1.84×10^{2}
Leu	12.3 ± 0.392	3.58×10	14.3 ± 0.308	1.83×10^{2}	12.6 ± 0.291	3.71×10	14.5 ± 0.272	1.74×10^{2}	10.9 ± 0.534	3.45×10	13.2 ± 0.815	1.19×10^{2}	10.5 ± 0.554	3.50×10	12.4 ± 0.278	1.33×10^{2}
Lys	26.7 ± 2.46	3.22×10^{3}	23.0 ± 2.21	1.50×10^{4}	28.2 ± 2.98	3.31×10^{3}	24.9 ± 2.13	2.10×10^{3}	20.5 ± 2.06	4.82×10^{2}	19.2 ± 2.47	5.06×10^{2}	19.9 ± 1.58	4.20×10^{2}	16.6 ± 0.356	3.96×10^{2}
Met	1.89 ± 0.214	1.34×10^{2}	4.52 ± 0.191	1.75×10^{2}	1.94 ± 0.178	9.57×10	4.71 ± 0.358	1.71×10^{2}	1.66 ± 0.0820	4.57×10	4.02 ± 0.241	7.38×10	0.462 ± 0.0510	4.21	1.11 ± 0.175	6.69
N_{1},N_{12}-Diacetylspermine	0.419 ± 0.0129	8.52×10^{2}	3.63 ± 0.112	1.18×10^{4}	0.419 ± 0.00609	9.32×10^{2}	3.61 ± 0.0468	5.19×10^{3}	0.449 ± 0.0627	5.17×10^{2}	4.10 ± 0.188	2.60×10^{3}	0.420 ± 0.0190	5.34×10^{2}	3.85 ± 0.0287	3.01×10^{3}
N_{1},N_{8}-Diacetylspermidine	0.415 ± 0.00819	1.26×10^{3}	3.59 ± 0.122	1.50×10^{4}	0.408 ± 0.00798	8.88×10^{2}	3.61 ± 0.0935	4.56×10^{3}	0.432 ± 0.0491	1.02×10^{3}	3.65 ± 0.166	2.60×10^{3}	0.394 ± 0.0187	3.71×10^{2}	3.37 ± 0.0445	2.94×10^{3}
N_{1}-Acetylspermidine	0.426 ± 0.0123	1.39×10^{2}	3.63 ± 0.127	1.00×10^{3}	0.436 ± 0.00439	3.08×10^{2}	3.65 ± 0.0451	1.19×10^{3}	0.446 ± 0.0621	1.35×10^{2}	3.60 ± 0.174	3.74×10^{2}	0.425 ± 0.0172	8.03×10	3.36 ± 0.0422	2.84×10^{2}
N_{1}-Acetylspermine	0.366 ± 0.0259	3.02×10^{2}	3.36 ± 0.158	7.59×10^{2}	0.386 ± 0.0283	3.37×10^{2}	3.67 ± 0.120	1.34×10^{3}	0.655 ± 0.0766	4.66×10^{2}	3.90 ± 0.180	1.43×10^{3}	0.401 ± 0.0135	2.13×10^{2}	3.65 ± 0.0615	9.40×10^{2}
N_{8}-Acetylspermidine	0.413 ± 0.0212	1.72×10^{2}	4.10 ± 0.141	7.26×10^{2}	0.411 ± 0.00779	2.12×10^{2}	4.09 ± 0.0623	9.37×10^{2}	0.376 ± 0.0511	7.14×10	3.25 ± 0.154	4.40×10^{2}	0.314 ± 0.0154	9.03×10	2.96 ± 0.0532	3.82×10^{2}
Phe	13.0 ± 0.233	3.82×10^{3}	14.7 ± 0.411	3.25×10^{3}	13.3 ± 0.348	3.60×10^{3}	15.5 ± 0.677	7.52×10^{3}	12.2 ± 0.745	1.82×10^{3}	13.2 ± 0.755	1.77×10^{3}	11.0 ± 0.514	1.29×10^{3}	13.1 ± 0.718	1.91×10^{3}
Pro	48.4 ± 1.84	8.77×10^{3}	46.8 ± 1.06	6.17×10^{3}	48.8 ± 1.32	7.17×10^{3}	47.5 ± 0.396	7.64×10^{3}	64.1 ± 6.03	3.55×10^{3}	61.7 ± 1.91	4.40×10^{3}	56.7 ± 3.37	5.04×10^{3}	56.6 ± 1.97	3.71×10^{3}
Spermidine	4.35 ± 0.440	9.17×10^{2}	9.00 ± 0.430	5.03×10^{2}	4.47 ± 0.450	9.04×10^{2}	9.40 ± 0.726	2.63×10^{2}	3.27 ± 0.375	4.29×10^{2}	7.70 ± 0.658	1.55×10^{3}	4.31 ± 0.535	5.19×10^{2}	8.48 ± 0.373	1.32×10^{3}
Spermine	2.95 ± 0.567	4.29×10^{2}	9.56 ± 0.768	1.16×10^{3}	2.26 ± 0.338	3.13×10^{2}	8.95 ± 0.563	6.06×10^{2}	1.47 ± 0.172	2.71×10^{2}	4.94 ± 0.505	8.92×10^{2}	2.29 ± 0.288	3.22×10^{2}	8.96 ± 1.15	1.12×10^{3}
Trp	1.91 ± 0.0534	3.20×10^{2}	4.88 ± 0.112	7.19×10^{2}	1.92 ± 0.0394	2.72×10^{2}	4.95 ± 0.0599	6.52×10^{2}	1.77 ± 0.101	1.03×10^{2}	4.44 ± 0.224	2.86×10^{2}	1.74 ± 0.0968	1.17×10^{2}	4.24 ± 0.0723	2.56×10^{2}
Tyr	24.8 ± 0.525	1.48×10^{3}	25.5 ± 0.704	1.64×10^{3}	24.4 ± 0.874	1.11×10^{3}	25.0 ± 1.45	7.08×10^{2}	20.4 ± 1.07	2.78×10^{2}	21.7 ± 1.08	1.22×10^{2}	18.7 ± 0.834	1.18×10	19.5 ± 0.162	1.58×10
Val	13.4 ± 1.29	9.25×10^{-2}	15.1 ± 0.990	9.75×10^{-2}	14.2 ± 1.44	8.00×10^{-2}	15.2 ± 1.29	9.25×10^{-2}	8.19 ± 2.96	3.28×10^{-1}	5.68 ± 1.03	4.20×10^{-1}	35.0 ± 1.66	6.10×10	33.6 ± 1.03	6.27×10
γ-Butyrobetaine	1.55 ± 0.0152	2.42×10^{3}	4.49 ± 0.119	5.64×10^{3}	1.59 ± 0.0215	2.56×10^{3}	4.47 ± 0.0460	5.33×10^{3}	1.30 ± 0.110	7.95×10^{2}	4.23 ± 0.221	2.48×10^{3}	1.26 ± 0.0571	5.77×10^{2}	3.90 ± 0.0465	1.08×10^{3}
Trimethylamine *N*-oxide	0.513 ± 0.00805	3.33×10^{2}	3.39 ± 0.105	1.23×10^{3}	0.518 ± 0.00632	2.67×10^{2}	3.37 ± 0.0466	1.60×10^{3}	0.475 ± 0.0485	7.64×10	3.02 ± 0.158	7.91×10^{2}	0.450 ± 0.0238	7.47×10	2.84 ± 0.0201	2.84×10^{2}
o-Acetylcarnitine	0.522 ± 0.0356	1.66×10^{2}	2.24 ± 0.0745	5.78×10^{2}	0.655 ± 0.0113	2.12×10^{2}	2.86 ± 0.0491	8.40×10^{2}	0.0687 ± 0.0402	1.29×10	0.236 ± 0.0610	1.25×10	0.497 ± 0.0249	5.89×10	2.07 ± 0.0214	4.60×10

[1]Saliva A and B spiked with 0.4 µM and 3.6 µM of standard mixture.

[2]Average and S.D. values (µM) were calculated by inter-day variance (n = 5).

Table S8. AUC of short-term storage sample data with noise added

Metabolite	Original AUC	Condition[1]	S.D.[2]	Median AUC	Lower AUC[3]	Upper AUC[3]
N_1-Acetylspermine	0.748	OI	0.007	0.749	0.706	0.794
		RT	0.003	0.750	0.706	0.794
N_1-Acetylspermidine	0.776	OI	0.009	0.778	0.774	0.780
		RT	0.009	0.778	0.774	0.781
Spermine	0.802	OI	0.082	0.797	0.771	0.824
		RT	0.530	0.749	0.688	0.810

[1]OI and RT indicate on ice and at room temperature, respectively.

[2]S.D. indicates standard deviation (µM) of computationally added noise.

[3]From 200 noise-added data sets, AUC values ranked at 2.5% and 97.5% are included.

Table S9. Long-term storage of AUC data with noise added

Metabolite	Original AUC	Ethanol[1]	Temperature	S.D.[2]	Median AUC	Lower AUC[3]	Upper AUC[3]
*N*1-Acetylspermine	0.748	no	-10°C	0.0057	0.750	0.705	0.791
		no	4°C	0.0068	0.748	0.705	0.792
		no	RT	0.0213	0.744	0.707	0.784
		added	-10°C	0.0032	0.745	0.712	0.791
		added	4°C	0.0057	0.750	0.697	0.793
		added	RT	0.0067	0.748	0.712	0.792
*N*1-Acetylspermidine	0.776	no	-10°C	0.0765	0.777	0.761	0.792
		no	4°C	0.0849	0.776	0.761	0.794
		no	RT	0.2113	0.767	0.730	0.796
		added	-10°C	0.1297	0.774	0.745	0.798
		added	4°C	0.0567	0.776	0.764	0.788
		added	RT	0.0438	0.777	0.767	0.787
Spermine	0.802	no	-10°C	0.0275	0.803	0.787	0.815
		no	4°C	0.6703	0.743	0.672	0.805
		no	RT	0.7627	0.730	0.655	0.803
		added	-10°C	0.0152	0.803	0.792	0.817
		added	4°C	0.0244	0.803	0.793	0.818
		added	RT	0.0271	0.803	0.790	0.817

[1]Saliva samples with ethanol (added) and without ethanol (no) addition.

[2]S.D. indicates standard deviation (μM) of computationally added noise.

[3]From 200 noise-added data sets, AUC values ranked at 2.5% and 97.5% are included
